# Temporal clustering analysis of endothelial cell gene expression following exposure to a conventional radiotherapy dose fraction using Gaussian process clustering

**DOI:** 10.1371/journal.pone.0204960

**Published:** 2018-10-03

**Authors:** Markus Heinonen, Fabien Milliat, Mohamed Amine Benadjaoud, Agnès François, Valérie Buard, Georges Tarlet, Florence d’Alché-Buc, Olivier Guipaud

**Affiliations:** 1 Department of Information and Computer Science, Aalto University, Aalto, Finland; 2 Institute for Radiological Protection and Nuclear Safety (IRSN), PSE-SANTE, SERAMED, LRMed, Fontenay-aux-Roses, France; 3 Institute for Radiological Protection and Nuclear Safety (IRSN), PSE-SANTE, SERAMED, Fontenay-aux-Roses, France; 4 LTCI, Télécom ParisTech, Université Paris-Saclay, Paris, France; International University of Health and Welfare School of Medicine, JAPAN

## Abstract

The vascular endothelium is considered as a key cell compartment for the response to ionizing radiation of normal tissues and tumors, and as a promising target to improve the differential effect of radiotherapy in the future. Following radiation exposure, the global endothelial cell response covers a wide range of gene, miRNA, protein and metabolite expression modifications. Changes occur at the transcriptional, translational and post-translational levels and impact cell phenotype as well as the microenvironment by the production and secretion of soluble factors such as reactive oxygen species, chemokines, cytokines and growth factors. These radiation-induced dynamic modifications of molecular networks may control the endothelial cell phenotype and govern recruitment of immune cells, stressing the importance of clearly understanding the mechanisms which underlie these temporal processes. A wide variety of time series data is commonly used in bioinformatics studies, including gene expression, protein concentrations and metabolomics data. The use of clustering of these data is still an unclear problem. Here, we introduce kernels between Gaussian processes modeling time series, and subsequently introduce a spectral clustering algorithm. We apply the methods to the study of human primary endothelial cells (HUVECs) exposed to a radiotherapy dose fraction (2 Gy). Time windows of differential expressions of 301 genes involved in key cellular processes such as angiogenesis, inflammation, apoptosis, immune response and protein kinase were determined from 12 hours to 3 weeks post-irradiation. Then, 43 temporal clusters corresponding to profiles of similar expressions, including 49 genes out of 301 initially measured, were generated according to the proposed method. Forty-seven transcription factors (TFs) responsible for the expression of clusters of genes were predicted from sequence regulatory elements using the MotifMap system. Their temporal profiles of occurrences were established and clustered. Dynamic network interactions and molecular pathways of TFs and differential genes were finally explored, revealing key node genes and putative important cellular processes involved in tissue infiltration by immune cells following exposure to a radiotherapy dose fraction.

## Introduction

Half of patients with tumors receive radiotherapy (RT) at some point during the course of their disease [1]. In combination with surgery and chemotherapy, RT achieves good results in terms of long-term survival and tumor cure in a variety of tumors. Although the latest generation devices deliver doses more and more precisely to the tumors, the therapeutic ratio of RT is still limited by normal tissue injury in organs at risk and by the radiation resistance of some tumors [2]. The vasculature plays a crucial role in tumor progression and in tumor sensitivity or resistance and is considered as a target in attempts to destroy tumors [3]. It also orchestrates wound healing in the case of radiation injury [3]. In the vasculature, the endothelium is considered as a promising target to improve the differential effect of RT in the future [4, 5].

The response of vascular endothelial cells to radiation exposure leads to a long-term radiation-induced dysfunction phenotype [6]. Conventionally, fractionated RT protocols deliver daily dose fractions of about 2 Gy. In a previous study, we analyzed primary human endothelial cells exposed to an ionizing radiation dose of 2 Gy to study temporal transcriptional perturbations from 0.5 to 21 days post-exposure [7]. This work consisted of developing a method for detecting time periods of differential gene expression using Gaussian processes (GPs) and a novel Bayesian likelihood ratio test. This allowed us to identify sets of differentially expressed genes in the different time periods after irradiation which, together with domain literature and gene enrichment analysis, led to insights into the dynamic response of endothelial cells to irradiation. We demonstrated that the method could well highlight phenomena already described in the response of cells to irradiation. Interestingly, the work suggested that endothelial cells may display an inflamed phenotype throughout RT, with possible effects on the vasculature of both normal tissues and tumors.

Here, we wanted to go deeper into the use of this dataset by a bioinformatics analysis of differentially expressed gene clusters. This new study has sought to establish a novel method to cluster time periods of statistically differentially expressed genes determined by our previous method of GPs and the Bayesian likelihood ratio test. This new method has been applied to our previously published dataset of real-time qPCR measurements of the transcriptional profiles of human umbilical vascular endothelial cell (HUVEC) genes following irradiation at 2 Gy.

With the advent of high-throughput measurements technologies, large-scale systems biology experiments are now routinely performed. Time series measurements of the transcriptomic state of cells can reveal important information on their inherently dynamic regulation and function. In this paper, we focus on the central task of determining the differentially expressed genes in a two-sample time series experiment [8–11]. To this end, both Bayesian and frequentist statistical tests have been proposed to estimate the significance of the difference between gene expression in two conditions, or the difference from steady-state kinetics [9, 11, 12], while [13] applies Fourier analysis to differentiate gene expressions. In the Bayesian approach, a Bayes factor between a null model and a differential model is often approximated by computing the likelihood ratios of the observed data against the competing time course gene expression models [14, 15]. A difference is declared if the data can be explained more confidently using the differential model. By computing the likelihood ratio for individual observation times, a differential test is produced over the observed time points [9]. However, it is highly desirable to be able to estimate differential expression smoothly over the time course even in the case of sparse or uneven measurements. To achieve a smoother estimation of differential expression, we propose two likelihood ratio tests that measure the expected data likelihood instead of the observed data likelihood. These can be evaluated naturally using probabilistic underlying expression models, or approximated using bootstrapping [16]. We considered the Gaussian process regression (GPR) models, which have been commonly applied to model time course gene expression [17–19] and which are an apt model for likelihood ratio estimation [9]. GPR models are a flexible class of non-parametric Bayesian models, which quantify the uncertainty of the underlying process estimates using Gaussian distributions [20]. GPR models of temporal gene expression have been extended with outlier detection [21], hierarchical replicate models and clustering [22], bootstrapping [16], missing data handling [13] and with ordinary differential equation (ODE) model integrations [23, 24]. Due to the GPR modeling, our approach is general to any kind of time series data, and supports any number of replicate measurements and time point distributions.

In this paper we defined several families of kernel functions between GPs and propose a novel clustering algorithm suitable for kernels between GPs. We propose to extend the method by considering kernels between derivatives of GPs as well as to model the rate of expression changes. We analyzed the performance of the proposed kernel families and applied the method to clustering of gene expression time series for irradiation of human endothelial cells. We sought results for predicted transcription factors (TFs) to gain insights into the biological relevance of the clustering as regards the response of endothelial cells to a conventional RT dose fraction (2 Gy), finally providing biological insight by cluster analysis.

## Materials and methods

### A description of the gaussian process kernel method

We first review the notions of GPs and kernels between distributions, and then present several families of kernels between GPs.

#### Primer on gaussian processes

First, we construct smooth probabilistic models of the measured gene expression trajectories over time from point measurements using GPs. Let $y=({y_{t}}_{1},\ldots ,{y_{t}}_{N})\in R^{N}$ be the vector of *N* noisy gene expression measurements $y_{t}\in R$ at input time points $T_{obs}=(t_{1},\ldots ,t_{N})\in R_{+}^{N}$. We assume that a true model *f*(*t*) explains the observations through

$$y_{t}=f(t)+{ℇ}_{t}$$

for some Gaussian isotropic and time-dependent noise model $E_{t}∼N(0,\omega_{t}^{2})$. We collect the time-dependent noise variances $\omega_{t_{1}}^{2},\ldots ,\omega_{t_{N}}^{2}$ into a diagonal covariance matrix Ω.

GPR is a Bayesian non-parametric and non-linear method for regression. A GP is a generalization of distributions to functions, where any subset of function evaluations is jointly Gaussian [20]. A GP $f_{⋆}∼GP(\mu_{⋆},\Sigma_{⋆})$ represents a distribution over function samples $f_{⋆}=f(t_{1}),{\ldots ,f(t}_{N_{⋆}})$ at time points $T=(t_{1},{\ldots ,t}_{N_{⋆}})\in R_{⋆}^{N}$ through the mean vector $\mu_{⋆}\in R^{N_{⋆}}$ and the covariance matrix $\Sigma \in R^{N_{⋆}\times N_{⋆}}$.

According to GPR modeling, we determine the function class by placing a Gaussian prior

$$f∼N(0,K_{TT})$$

over the true model *f*(*t*), where *K*_*TT*_ is a covariance, or more generally, a positive semi-definite kernel matrix between time points *T*_*obs*_ × *T*_*obs*_. We are interested in learning the GP given the data y and the function prior, which results in a posterior distribution $f_{⋆}|y∼N(\mu_{⋆},\Sigma_{⋆})$ defined by

$$\mu_{⋆}=K_{⋆T}{{(K}_{TT}+\Omega )}^{-1}y$$

$$\Sigma_{⋆}=K_{⋆⋆}-{K_{⋆T}{(K}_{TT}+\Omega )}^{-1}K_{T⋆}$$

where $K_{⋆T}=K_{T⋆}^{T}$ is the kernel *K* over *T*_⋆_ × *T*_*obs*_.

The posterior of the true model can be visualized by the mean model *μ*_⋆_ along with 95% confidence intervals $\pm 1.96\sqrt{diag\Sigma_{⋆}}$. However, if we are interested in sampling from the estimated model with observational noise Ω, we use the distribution $y_{⋆}\equiv y_{⋆}|f_{⋆}∼N(\mu_{⋆},\Sigma_{⋆}+\Omega )$ as the complete noisy kinetic model of the gene expression [16].

The kernel choice *K*(*t*,*t*′) plays an important role in determining the function space learned by the GP. The Gaussian kernel *K*(*t*,*t*′) = exp(−‖*t* − *t*′‖^2^/2*l*^2^) is often used as a “default” kernel because of its simplicity, which naturally gives high covariance for close time points, resulting in smooth regression models. However, the Gaussian kernel is a function of *t* − *t*′, and hence *stationary*. For non-stationary dynamics, we opt for the non-stationary Gaussian kernel [7]

$$K_{l}(t,t^{\prime })=\sigma_{f}^{2}\;\exp\;\left(-{\left(\frac{t}{l(t)}-\frac{t^{\prime }}{l(t^{\prime })}\right)}^{2}\right)$$

where we can choose a simple log-transform *l*(*t*) = log(*t*) or a parametric time-transformation *l*(*t*) =*l* − (*l* − *l*_*min*_)*e*^−*ct*^. The three hyperparameters are: maximum length scale *l*, minimum lengthscale *l*_*min*_ (at time *t* = 0), and the curvature *c* controls how fast the function *l*(*t*) approaches its maximum value. We assume that the data are normalized such that perturbation occurs at time 0.

The GPR framework provides a natural way to learn the hyperparameters *θ* = (*σ*_*f*_,*l*,*l*_*min*_,*c*) of the kernel *K*_*l*_. In a Bayesian model inference we would marginalize over the hyperparameters and the models implied by them. Due to computational tractability, we instead learn hyperparameters against the marginal log likelihood (MLL)

logp(y|T,θ)=log∫p(y|f,T)p(f|θ)df (1)

which follows $y∼N\left(0,K_{TT}+\theta \right)$ giving a log likelihood $-\frac{1}{2}y^{T}{\left(K_{TT}+\theta \right)}^{-1}y-\frac{1}{2}log|K_{TT}+\theta |-\frac{N}{2}log2\pi$. We optimize the parameters *θ* by gradient descent over Eq (1) with L-BFGS. We set the noise model to the replicate measurement variances. Alternatively, the noise model can be learned also against the marginal log likelihood [22], which, however, leads to an intractable inference if a varying noise model is considered.

#### Gaussian process kernels

We are interested in defining kernel functions between two GPs to be used for subsequent unsupervised or supervised learning. Let y∼GP(μ,Σ) and y′∼GP(μ′,Σ′) be GPs with means and covariances defined through N-dimensional multivariate normal distribution instantiations, where $\mu ,\mu \prime \in R^{N}$ and $\Sigma ,\Sigma \prime \in R^{N\times N}$. The distributions y and y′ represent GPs over *N* time points (*t*_1_,…,*t*_*N*_). The more time points we utilize, the more accurate the GPs are.

While kernels between distributions, such as probability product kernels [25], Kullback-Leibler kernel [26] or Fisher kernels [27] have been defined, they are not directly applicable to stochastic processes. They do however offer a promising path towards GP kernels. Realizations of GPs are in practice N-dimensional multivariate normals that represent the process with higher N giving a more accurate realization of the GP. The kernel function between GPs should reflect this property and converge towards the true kernel value when N approaches infinity. We call a kernel *GP* − *convergent* if this holds. The distribution-based kernels listed above converge to zero as we increase *N*, unless the objects are identical.

We propose three families of GP kernels, the overlap coefficient (OVL) kernel *K*_*OVL*_, and *GP* − *convergent* variants of the probability product *K*_*PP*_ and the symmetric Kullback-Leibler kernels *K*_*KL*_. We are interested in defining a kernel

K(y,y′)≡K(p,p′)

in following the notation of [25], where *p* is the density function of the corresponding MVN distribution. Comparing N-dimensional MVN distribution is numerically intractable, and hence we define a GP kernel as a weighted sum over the marginalized distributions

$$K(p,p\prime )\equiv \int_{R}K(p_{t},{p\prime }_{t})dt$$

where *p*_*t*_ is the marginalized Gaussian density at time *t*. This simplification entails only considering the diagonal variance diag Σ, which corresponds to the marginalized variances of the GP over time, which are commonly used to represent the model. Finally, we propose to enhance the kernel by taking a weighted mean according to the time interval lengths $\Delta t_{i}=\frac{1}{2}(t_{i+1}-t_{i-1})$ at all time points (note that first and last time points are handled as special cases) over a regularly spaced sample *T* = (*t*_1_,…,*t*_*N*_) as

$$K_{N}(p,p^{\prime })=\frac{1}{\Delta T}\sum_{i=1}^{n}\Delta t_{i}K(p_{i},{p^{\prime }}_{i})$$ (2)

where Δ*T* is the time window length and Δ*t* is the time window length length. The kernel has the property lim_n→∞_ *K*_*N*_(*p*,*p*′) = *K*(*p*,*p*′).

#### Probability product kernels

In [25] a probability product kernel is defined as

$$K(p,p\prime )\equiv \int_{R^{N}}p{\left(z\right)}^{\rho }p\prime {\left(z\right)}^{\rho }dz$$

over various distributions. Two interesting special cases arise: with *ρ* = 0.5 the kernel turns into a *Bhattacharyya* kernel [28]

$$\int_{R^{N}}\sqrt{p(z)}\sqrt{p\prime (z)}dz$$

which is naturally normalized such that *K*(*p*_*i*_,*p*′_*i*_) = 1; and *ρ* = 1 gives the *expected likelihood* kernel

$$\int_{R^{N}}p(z)p^{\prime }(z)dz=E_{p}[p^{\prime }(z)]=E_{p\prime }[p(z)]$$

which is the expectation of one distribution under the other. A closed form solution of the one-dimensional case $K(p_{i},{p^{\prime }}_{i})=\int_{R}p_{i}(z){p^{\prime }}_{i}(z)dz$ for the Bhattacharyya case *ρ* = 1/2 is

$$K_{BH}(p_{i},{p^{\prime }}_{i})=\sqrt{\frac{2\sigma_{i}{\sigma^{\prime }}_{i}}{\sigma_{i}^{2}+{\sigma^{\prime }}_{i}^{2}}}\exp\left(-\frac{1}{4}(\frac{\mu_{i}^{2}}{\sigma_{i}^{2}}+\frac{{\mu^{\prime }}_{i}^{2}}{{\sigma^{\prime }}_{i}^{2}}-\frac{\sigma_{i}^{2}{\sigma^{\prime }}_{i}^{2}(\frac{\mu_{i}}{\sigma_{i}^{2}}+\frac{{\mu^{\prime }}_{i}}{{\sigma^{\prime }}_{i}^{2}})}{\sigma_{i}^{2}+{\sigma^{\prime }}_{i}^{2}})\right)$$ (3)

and for the expected likelihood case *ρ* = 1

$$K_{EL}(p_{i},{p^{\prime }}_{i})=\frac{1}{\sqrt{{2\pi (\sigma }_{i}^{2}+{\sigma^{\prime }}_{i}^{2})}}\exp\left(-\frac{1}{2}\frac{{\left(\mu_{i}-{\mu^{\prime }}_{i}\right)}^{2}}{\sigma_{i}^{2}+{\sigma^{\prime }}_{i}^{2}}\right)$$ (4)

The kernel is SDP and N-convergent. Adding new time points during the GP only changes the similarity value if the new time points encode new information. We note that an alternative formulation of weighted geometric mean would result in a non-SDP kernel.

#### Kullback-Leibler kernel

Kullback-Leibler divergence between two MVNs is

$$D_{KL}(p\|p\prime )=\int_{R^{N}}\log\frac{p(z)}{p\prime (z)}p(z)dz,$$

and has an analytical solution. Considering only diagonal covariance results in

$$D_{KL}(p\|p\prime )=\frac{1}{2}\sum_{i=1}^{n}\left(\frac{{\left(\mu_{i}-{\mu \prime }_{i}\right)}^{2}}{{\sigma \prime }_{i}^{2}}+\frac{{\sigma \prime }_{i}^{2}}{\sigma_{i}^{2}}-1-\log\frac{\sigma_{i}^{2}}{{\sigma \prime }_{i}^{2}}\right).$$

The Kullback-Leibler divergence is not symmetric, and hence we define a two-way symmetric KL divergence

$$\begin{array}{l} D_{KL}(p,p\prime )=D_{KL}(p\|p^{\prime })+D_{KL}(p\prime \|p)\\ \;=\frac{1}{2}\sum_{i=1}^{n}\left(\frac{{\left(\mu_{i}-{\mu \prime }_{i}\right)}^{2}+\sigma_{i}^{2}}{{\sigma \prime }_{i}^{2}}+\frac{{\left(\mu_{i}-{\mu \prime }_{i}\right)}^{2}+{\sigma \prime }_{i}^{2}}{\sigma_{i}^{2}}-2\right)\\ \;=\frac{1}{2}\sum_{i=1}^{n}D_{KL}(p_{i},{p^{\prime }}_{i}) \end{array}$$

as a basis for the KL kernel

$$K_{KL}(p,p^{\prime })=\exp\left({-\alpha D}_{KL}(p,p\prime )+\beta \right)$$

with *α* being a scaling parameter and *β* a shift. The KL kernel converges to zero while N grows. The *α* can be used to lessen the effect (e.g. setting *α* = *N*), but using small *α* leads to the kernel becoming numerically non-SDP.

We adapt the KL kernel into a GP kernel by taking the weighted mean of the divergence according to the time intervals

$$\begin{array}{l} D_{KL}(p,p\prime )=\exp\;\left(-\frac{\alpha }{2\Delta T}\sum_{i=1}^{n}{\Delta t_{i}D}_{KL}(p_{i},{p\prime }_{i})+\beta \right)\\ \;=\exp\;\left(-\frac{\alpha }{2\Delta T}\sum_{i=1}^{n}\Delta t_{i}(\frac{{\left(\mu_{i}-{\mu \prime }_{i}\right)}^{2}+\sigma_{i}^{2}}{{\sigma \prime }_{i}^{2}}+\frac{{\left(\mu_{i}-{\mu \prime }_{i}\right)}^{2}+{\sigma \prime }_{i}^{2}}{\sigma_{i}^{2}}-2)+\beta \right). \end{array}$$ (5)

#### Overlapping coefficient kernel

We propose another distribution similarity that measures the overlap between two distributions [29]. An OVL between densities *p*(*z*) and *p*′(*z*) is

$$K_{OVL}(p,p^{\prime })=\int_{R^{N}}\min\left\{p(z),p\prime (z)\right\}dz$$ (6)

$$=1-\|p-p^{\prime }\|\in [0,1]$$ (7)

and is a valid kernel as the norm of the distance of the distributions [30].

The OVL naturally measures both the shape and GP uncertainties. The similarity generalizes into any distributions. The overlap measures the volume of the overlapping region of the distributions, or the area under the overlapping curve for one-dimensional distributions.

Parametric and non-parametric estimation frameworks for computation of OVL have been proposed [30, 31]. However, for our purposes, the overlap can be computed analytically for one-dimensional Gaussians, and hence we propose to compute the OVL for diagonalized covariances as

$$\begin{array}{l} K_{OVL}(y,y^{\prime })=\frac{1}{\Delta T}\sum_{i=1}^{n}{\Delta t_{i}S}_{OVL}(p_{i},{p^{\prime }}_{i})\\ \;=\frac{1}{\Delta T}\sum_{i=1}^{n}\Delta t_{i}\int_{R}\min\left\{p_{i}(z),p_{i}\prime (z)\right\}dz. \end{array}$$ (8)

The overlap between two Gaussians decomposes into a minimum of at most three intervals, each of which can be computed using the cumulative density of the smaller density. When the two Gaussians have equal variances, only two intervals emerge. When also the means are equal, only a single interval emerges. We handle these as special cases. Therefore, the three intervals have two points *z*_1_ and *z*_2_ of equal density, which are the solutions to the square equation

$$\left(\frac{1}{{2\sigma }^{2}}-\frac{1}{{2\sigma \prime }^{2}}\right)z_{i}^{2}-\left(\frac{\mu^{\prime }}{{\sigma^{\prime }}^{2}}-\frac{\mu }{\sigma^{2}}\right)z_{i}+\left(\frac{\mu^{2}}{{2\sigma }^{2}}-\frac{{\mu^{\prime }}^{2}}{{2\sigma^{\prime }}^{2}}+\log\frac{\sigma }{\sigma^{\prime }}\right)$$

The overlap kernel is then

$$\begin{array}{l} K_{OVL}(p_{i},{p^{\prime }}_{i})=\int_{R}\min\left\{p(z),p\prime (z)\right\}dz\\ \;=\min\left\{F(z_{1}),F\prime (z_{1})\right\}+\min\{F(z_{2})-F(z_{1}),F\prime (z_{2})-F\prime (z_{1})\}+\min\left\{1-F(z_{2}),1-F\prime (z_{2})\right\} \end{array}$$

where *F* is the cumulative distribution function of a Normal.

#### Difference of similarities

The four similarities have different interpretations. The overlap similarity measures the average volume of the overlapping or shared distribution over time, and is naturally normalized between 0 and 1. The two probability product kernels measure the geometric mean of two distributions. We note that in general one cannot retrieve the Bhattacharyya kernel by normalizing the expected likelihood kernel. The OVL kernel is a lower bound of the Bhattacharyya kernel. Finally, the Kullback-Leibler kernel is the expectation of the log difference between the two densities over the other one, and has well-known theoretical interpretations of information.

#### Derivative gaussian processes

A derivative of a GP is another GP, as a derivative is a linear operation [32–34]. In a regression setting, given observations y at time points *T*, the GP $N_{\vartheta }(\mu_{⋆}\prime ;\Sigma_{⋆}\prime )$ of the derivative *f*_*ϑ*_ of the estimated true function *f*(*t*) at target time points *T*_⋆_ is defined as [35]

$$\mu_{⋆}^{\prime }=\frac{\vartheta K_{⋆T}}{\vartheta {KT}_{⋆}}{\left(K_{TT}+\Omega \right)}^{-1}y$$

$$\Sigma_{⋆}^{\prime }=\frac{\vartheta^{2}K_{⋆⋆}}{\vartheta^{2}T_{⋆}}-\frac{K_{⋆T}}{\vartheta T_{⋆}}{\left(K_{TT}+\Omega \right)}^{-1}y\frac{K_{T⋆}}{\vartheta T_{⋆}}.$$

Utilizing derivative GPs allows comparison of the change of variable over time in addition to comparing the variable values directly. A kernel between derivative GPs is in a normal fashion used as *K*(*y*_*ϑ*_,*y*′_*ϑ*_), e.g. *K*_*KL*_(*y*_*ϑ*_,*y*′_*ϑ*_). A mixture kernel

$$K(y,y\prime )+(1-\alpha )K(y_{\vartheta },{y\prime }_{\vartheta })$$

compares both the variable and its rate of change with ratio *α*, i.e. *α* = 0.5.

#### Spectral k-means—Clustering

Spectral clustering algorithms are a special class of clustering algorithms that are based on the graph Laplacians of similarity matrixes between objects [36] (see [37] for a review). The data are mapped into the eigenspace determined by the first *k* eigenvectors of the Laplacian

L=D−K

where K is the similarity measure or a kernel, and D is a diagonal matrix with *D*_*ii*_ = ∑_*j*_*K*_*ij*_. The normalized graph Laplacian is *L*_*norm*_ = *D*^−1/2^*LD*^−1/2^. This translation maximizes the separation of the components of the underlying structure [37]. The clustering is achieved through standard *k*-means over the translated points. However, this leaves the procedure vulnerable to outliers and noise. We propose to couple the Laplacians with an outlier-resistant *k*-means variant, denoted as *k*-means—[38].

The k-means consists of three iterative steps for some initial clustering: (i) computing the *l* most distant points from the nearest cluster centers, (ii) determining the closest cluster centers for the remaining points, and (iii) computing new cluster centers as their mean. To couple the method with spectral clustering, we apply the weighting scheme of [36] in the spectral domain, or over the principal components of the graph Laplacian, as detailed in the algorithm presented in S1 Fig.

### Dataset

We used the dataset we generated and published previously [7]. This dataset corresponds to real-time qPCR measurements of the transcriptional profiles of 301 human umbilical vascular endothelial cell (HUVEC) genes following irradiation at 2 Gy. Briefly, in the previous study, transcriptional profiles of 301 genes of HUVECs were measured with real-time qPCR under control conditions and with a single irradiation dose of 2 Gy (case) at 0 h with measurements *T*_*obs*_ at 12 h, 1, 2, 3, 4, 7, 14 and 21 days. Gene expression assays were performed using a panel of premade TaqMan low-density array gene signature (angiogenesis, inflammation, apoptosis, immune response and protein kinase) (Applied Biosystems). Experiments were performed in triplicate for each time point of the time course. GPR models were learned for each gene under both conditions over prediction time points *T*_⋆_ that cover smoothly days 0 to 24 [7].

### Clustering with the new gaussian process kernel method

We clustered the gene expression curves by the OVL kernel and the outlier-resistant spectral clustering as described in the Results and Discussion section.

### Transcription factor enrichment

The MotifMap system [39, 40] (http://motifmap.ics.uci.edu/) was employed to obtain TF motifs present within promoters (-1000 to +1000 bp relative to transcription star site; TSS) and predictions of candidate regulatory elements with a Bayesian branch length score (BBLS) score of at least 1 and a false discovery rate (FDR) of 0.1.

### Data visualization

The differential genes, gene clusters and putative gene-associated TFs were visualized as an “eye diagram” using the published code available from http://www.cis.hut.fi/projects/mi/software/ismb09 [41].

### Density plots

The smoothed histograms were generated using the ‘ks’ R-package [42] which is a plug-in estimator Hpi bandwidth selection criterion.

### Clustering of transcription factor profiles

The approach adopted here for the transcription factor profile clustering stems from a field of statistics known as functional data analysis (FDA) [43]. For each gene i among the 47 genes of interest, the time-varying number of TFs, Y_ij_, measured during 21 days is considered as a realization of a random time-dependent functional process X_i_ with

$$Y_{ij}=X_{i}(t_{j})+\varepsilon_{ij}$$

Where t_j_ is the j^th^ day and {ε_ij_} are a collection of independent and identically distributed random variables with mean 0 and variance σ^2^.

The two-stage clustering method of these functional data starts with a dimension reduction step using functional principal component analysis (FPCA) followed by a second step which consists in clustering the scores obtained using a hierarchical complete-linkage algorithm. More precisely, the goal of the FPCA is quite the same as its multivariate counterpart since its aims is to succinctly describe the TF time variations that explain the most variability. Thus, the FPCA represents each TF profile in term of the Karhunen-Loève decomposition [44]

$$X_{i}(t)=\bar{X}(t)+\sum_{k\geq 1}\rho_{ik}\times \phi_{k}(t)$$

where $\bar{X}(t)=\frac{1}{47}\sum_{i=1}^{47}X_{i}(t)$ is the common mean, (ϕ_k_) the eigenfunctions which exhibit, in an optimal way according to a variance criterion, the main modes of variation of the TF profiles relative to $\bar{X}$. (ρ_ik_) are uncorrelated random effect variables (scores) with mean 0 and variances λ_k_ (eigenvalues) in descending order to be interpreted as the contribution of k^th^ variation mode to the total explained variance. Finally, (ϕ_k_) are the functional principal components (FPC) or eigenfunctions which are orthogonal according to the inner product 〈u,v〉 = ∫u(t)v(t)dt.

As usually done in the multivariate case, each TF profile was normalized by dividing the values of each function by their standard error to account for differences in degrees of magnitude among the TF time variation functions.

The functional principal component scores (FPCS) were calculated using the Matlab package PACE [45] and the FPCS number included in the hierarchical complete-linkage algorithm was selected according to the percentage of explained variance (here 95%), which is a usual criterion in FPCA.

### Pathway analysis of differentially expressed genes

Pathway and sub-network enrichment analyses were performed using the web version of the software Pathway Studio (Mammalian, ChemEffect, DiseaseFX, version 11.2.5.9, updated Oct 22, 2016) from Elsevier [46]. Names and expression ratio values of the differentially expressed genes and associated TFs were imported into the Pathway Studio. The data input was queried against the Pathway Studio knowledge base for biological interactions. Proteins mapped to the knowledge base were used to build protein networks. Interaction networks were added that included “radiation” as a treatment.

### Endothelial cell culture, irradiation procedure, RNA isolation and RT real-time PCR

This section describes the experiments performed to collect expression data on endothelial cells exposed to either a single dose of 20 Gy or ten fractionated doses of 2 Gy. HUVECs from Lonza were cultured in EGM-2-MV medium at 37°C with 5% CO_2_. Confluent cells were irradiated at passage 3 with a cesium-137 source (IBL 637, CisBio; dose rate 1 Gy/min). For dose-fractionation experiments, cells were irradiated with five fractions of 2 Gy per week for two weeks (including one weekend break). For long-term experiments (14 and 21 days post-irradiation, and dose-fractionation experiments), culture medium was changed every week. Total RNAs were prepared with the total RNA isolation kit (Rneasy Mini Kit, Qiagen) at day 0.5, 1, 2, 3, 4, 7, 14, 21 post-irradiation at a single dose of 20 Gy, and at day 21 after the first fraction of 2 Gy and day 21 after the last fraction of 2 Gy for dose-fractionation experiments. Total RNA integrity was analyzed using Agilent 2100 and after quantification on a NanoDrop ND-1000 apparatus (NanoDrop Technologies). Reverse transcription was performed using the High Capacity Reverse Transcription Kit (Applied Biosystems) according to the manufacturer’s instructions. Gene expression assays were performed using a panel of premade TaqMan low-density array (TLDA) gene signature array (angiogenesis, inflammation, apoptosis, immune response and protein kinase) (Applied Biosystems). cDNA (400 ng) per sample was loaded onto the port of each gene signature array card and PCR was performed with the ABI PRISM 7900 Sequence detection system (Applied Biosystems). Analyses were conducted according to the procedure previously described in detail [47]. Data Assist software (Applied Biosystems) was used to determine fold changes, with fixed criteria: a maximum allowable Ct value at 37 was fixed and maximum Ct values were not included in calculations. Normalization was performed using a global normalization method on a per sample basis [48]. Experiments were performed in triplicate for each time point of the time course. Data are given as means ± SD. Student’s *t*-test *p*-values were adjusted using the Benjamini-Hochberg false discovery rate method using Data Assist software, and an adjusted *p*-value less than 0.05 was applied to select statistically differentially expressed genes.

## Results and discussion

**Fig 1:**
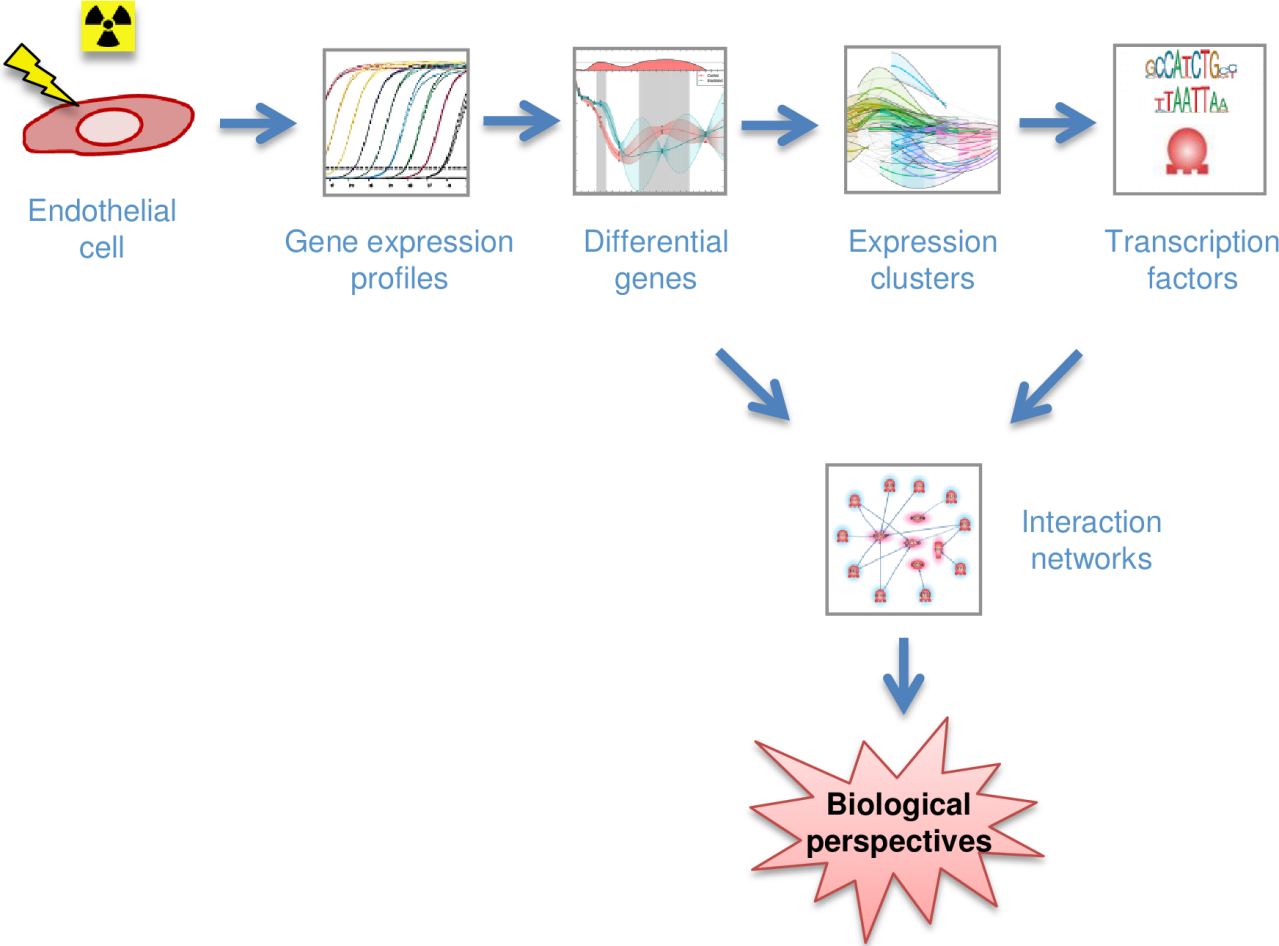
Overview of the methodology to study the transcriptional response of endothelial cells to a conventional radiotherapy dose fraction. Human umbilical vein endothelial cells (HUVECs) were used as model of primary human endothelial cells. HUVECs were irradiated at 2 Gy using a cesium-137 source of ionizing radiation. Time-course analysis of transcription profiles of about 450 genes was performed by RT-qPCR from 12 hours to 3 weeks post-irradiation and time windows of differential genes were determined using GPs as in ref. [7]. Temporal profiles of gene expression were then clustered with the new method presented in this paper, and regulatory motif sites were searched using the MotifMap system to propose putative TFs responsible for the expression of these genes. Finally, the data were analyzed using Pathway Studio software to explore network interactions and molecular pathways and to allow biological perspectives.

**Fig 2:**
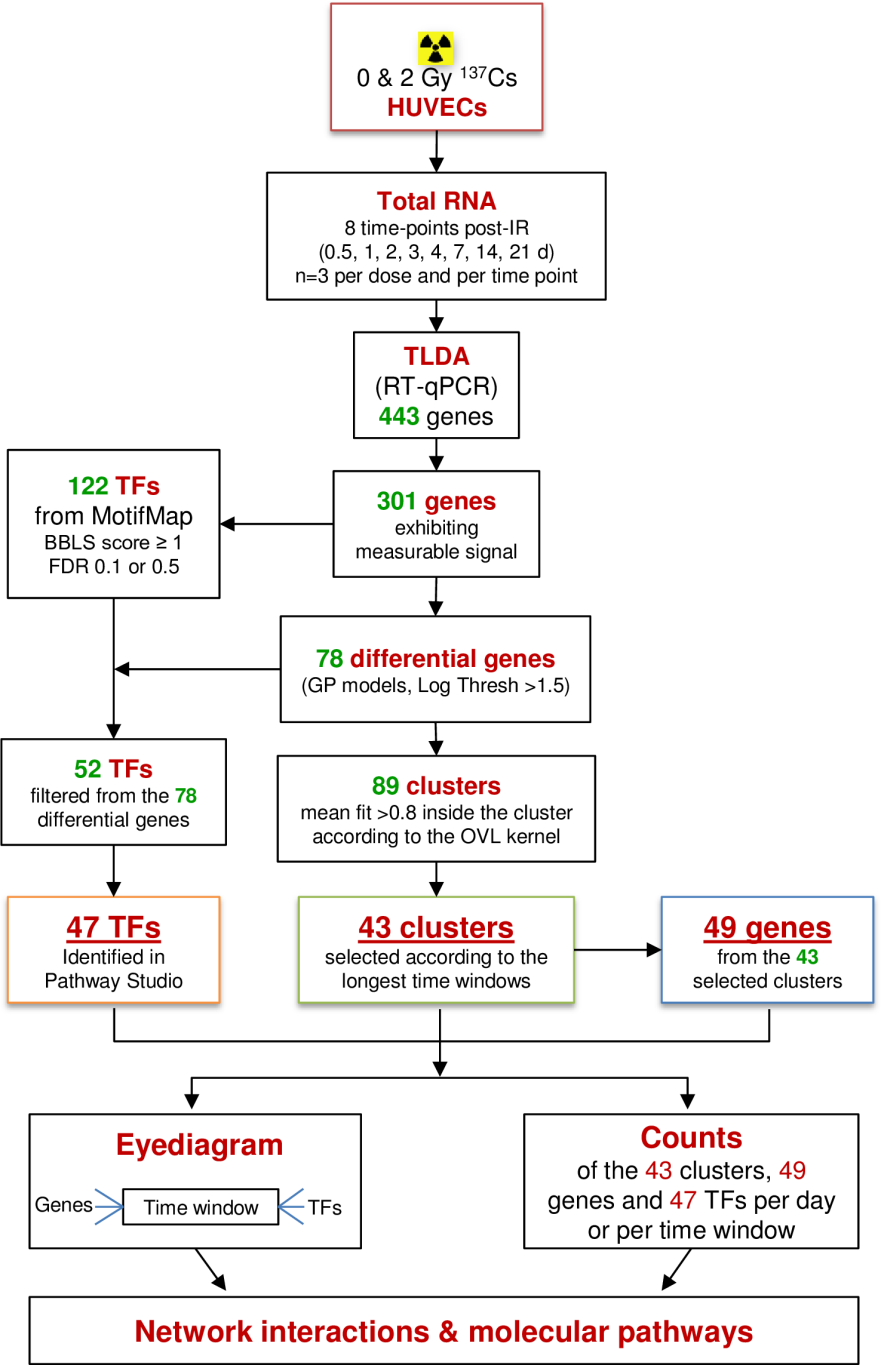
Workflow of data analysis. Of the 450 genes measured from 12 hours to 3 weeks post-2 Gy irradiation, 301 were reliably detected. Analysis of the temporal expression profiles identified 78 differential genes that finally gave rise to the definition of 43 clusters of expression with 49 genes, and the detection of 47 putative transcription factors (TFs). At the end, occurrences of TFs and differential genes allowed us to propose biological perspectives by analysis of molecular pathways and network interactions.

We experimented with the proposed kernels and the clustering method and then applied them to real data. To gain insights into the biological relevance of the clustering as regards the response of endothelial cells to a conventional RT dose fraction (2 Gy), (i) we clustered the gene expression curves by the OVL kernel and the spectral clustering, (ii) we searched for putative TFs associated with the clustered differential genes and (iii) we searched for pathway relationships between TF, gene entities and the term “radiation”. Fig 1 shows the overall methodology used in this work and Fig 2 displays the workflow of data analysis, from irradiation of cells to clustering and network interaction analysis of genes and TFs. The new method and the results (both on simulated data and real data) are presented below.

### Simulated data

First, we employed simulated clustering data—generated by GP models—to analyze which kernel is best, and which clustering method is best. Afterwards, we simply employed the OVL kernel and outlier-resistant spectral method to real data.

We generated simulated clusters by generating 50 GPs $N(\mu_{i},\Sigma_{i})$, *i* = 1,…,50 where

$$\mu_{i}∼N(0,\sigma_{f}^{\mu }K_{l^{\mu }})$$

$$\Sigma_{i}=\sigma_{f}^{\Sigma^{2}}K_{l^{\Sigma }}$$

with

σfμ∼Gam(4,14)

σfΣ∼Gam(2,12)

lμ∼Gam(4,32)

lΣ∼Gam(4,32)

and where *K*_*l*_ is a Gaussian kernel with length scale *l*. Hence, each simulated cluster is represented as a GP whose mean is a sample from another GP, and whose covariance is a kernel matrix defined by a *sigma*_*f*_ and *l* parameters sampled from Gamma distributions. We sampled 300 time series from these 50 GP clusters, while also sampling 100 independent time series, which represent outliers. Hence, the true outlier ratio is 25%.

#### Kernel comparison

We compared the performance of the four kernels on simulated data with 400 time series from 50 true clusters. We generated the simulated data, learned the GP models, computed the kernel matrices and applied standard spectral clustering. We repeated the experiment 10 times, and report average results. S2 Fig indicates the ROC curves for F1, recall and precision for the four kernels over the number of clusters derived from the spectral clustering. The true number of clusters is 50 with true outlier ratio of 25%. The OVL and Bhattacharyya kernel perform consistently well, with areas under F1 curves of 0.25 and 0.26, respectively. The KL kernel performs poorly (AUC 0.19), and the EL kernel seems to be a slightly less robust version of the BH (AUC 0.23). The precision and recall results are similar. For the rest of the paper, we then chose the OVL kernel as a robust GP kernel due to its better interpretability compared to the Bhattacharyya kernel.

#### Clustering method comparison

S3 Fig indicates the precision, recall and F1 of the three clustering methods on the simulated data using the OVL kernel. We compare standard spectral clustering combined with (i) k-means, (ii) outlier-resistant k-means—and (iii) EM clustering. The standard spectral clustering assigns all curves to some cluster and does not handle outliers. The k-means—is a k-means variant with *l* curves furthest away from the clusters left out at each iteration. The EM clustering is a probabilistic Gaussian mixture model, where an outlier distribution is maintained. S3 Fig shows that both standard k-means clustering and the outlier-resistant k-means—perform well with F1 AUCs of 0.25 and 0.25. However the outlier-resistant variant has higher precision, as expected, while having a lower recall. For biological studies, trading of the recall for higher precision is in general beneficial to reduce the number of false positives. Hence, we chose the spectral k-means—as our clustering method of choice for the real clustering experiments.

### Gene expression temporal clustering

**Fig 3:**
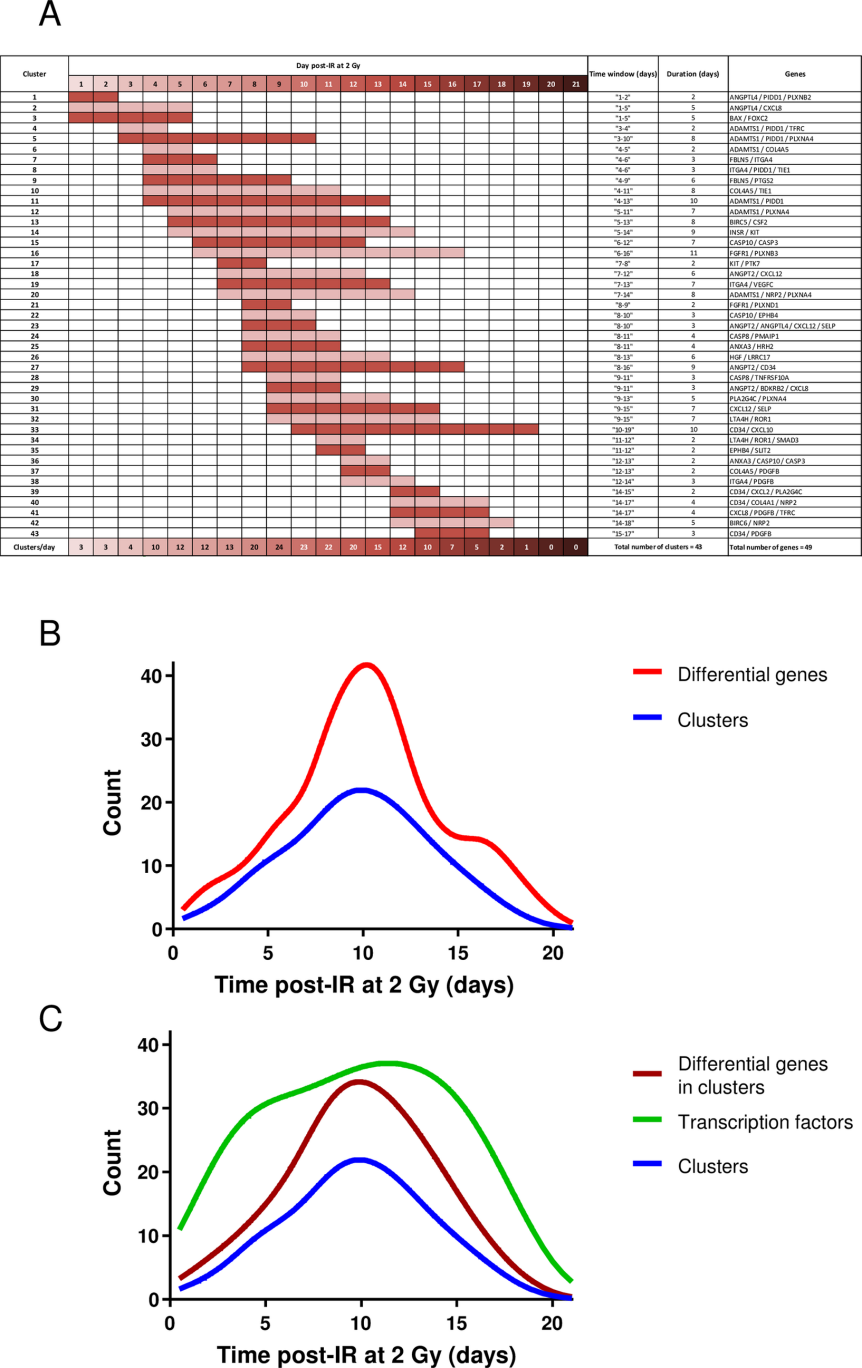
Cluster and transcription factor kinetics. (A) Visualization of the duration of each of the 43 clusters over the 3-week study period, and identities of genes within each cluster. (B) Number of differential genes and different clusters at each time post-irradiation over the 3-week study period. (C) Number of different clusters, differential genes in the different clusters, and predicted TFs putatively involved in the molecular response of endothelial cells at each time post-irradiation over the 3-week study period.

**Fig 4:**
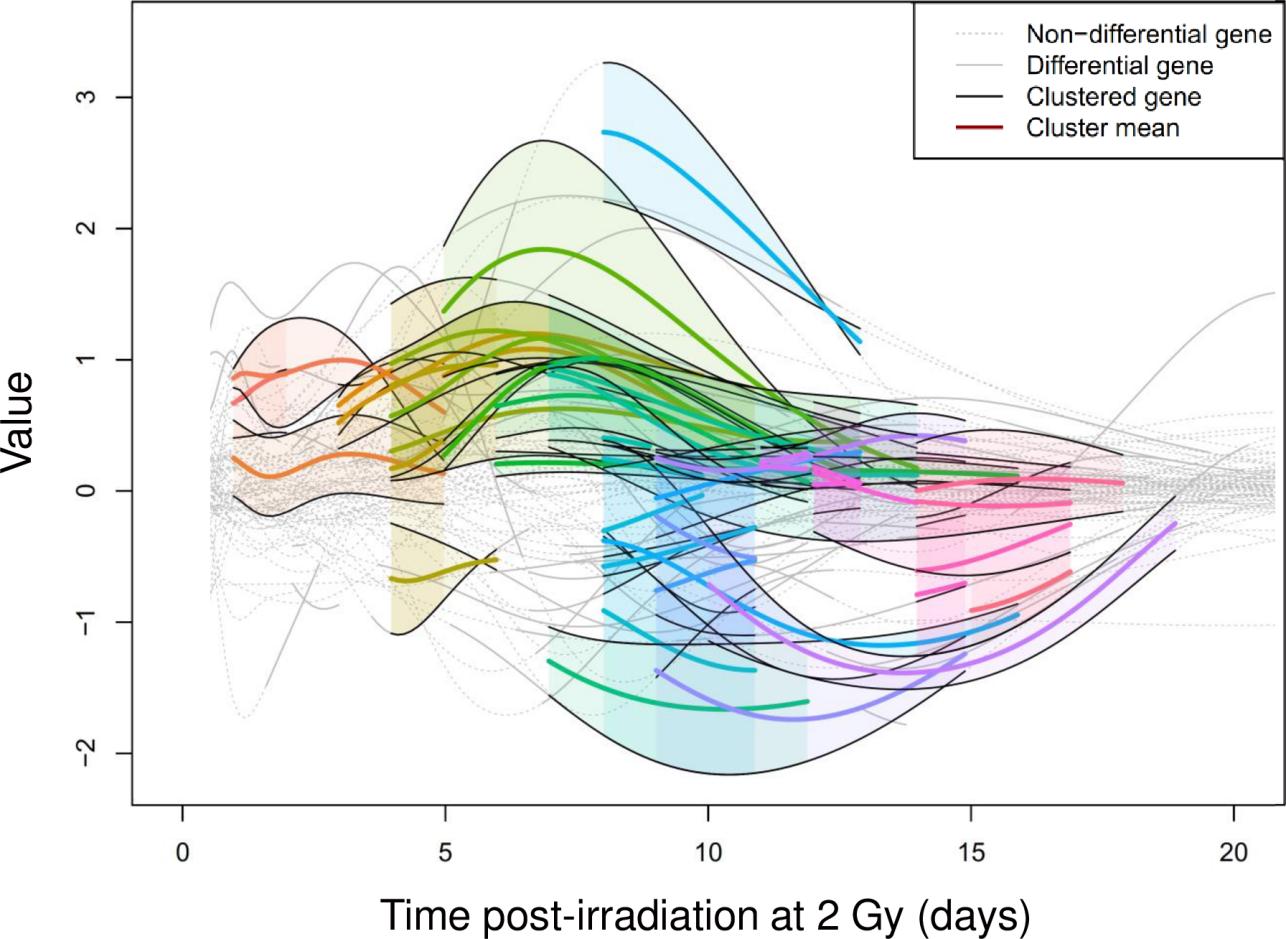
Cluster visualization. The 43 cluster means of differential genes were determined using a ratio threshold of 1.5 and a minimum cluster kernel similarity of 0.75. Clusters are displayed as colored curves and clustered genes as black curves over the 3-week study period. Gene expression profiles between the control and the irradiated samples are plotted as continuous gray curves in periods when the genes were differentially expressed and as a gray dotted curve when the genes were not differential.

We clustered the gene expression curves by the OVL kernel and the spectral clustering. We constructed the different curves and clustered the gene expression curves by the proposed methods. We retrieved a set of temporal clusters corresponding to similar profiles. We clustered both difference curves as well as the irradiated curves. As an application, we used the dataset we generated and published previously [7]. This dataset corresponds to real-time qPCR measurements of the transcriptional profiles of 301 HUVEC genes following irradiation at 2 Gy. In the present work, the published dataset was used to cluster the gene expression curves by the proposed new OVL kernel and the spectral clustering method, which had never been done before. The 301 gene expression profiles were obtained from the GP models computed earlier [7]. We chose all genes that have a differential expression using a log-threshold of 1.5 for at least 24 hours, resulting in 78 genes. We clustered these 78 genes over 154 time windows between 0.5 and 21 days, with all time window lengths between 1 and 8 days, i.e. clustering at intervals [0,1], [0,2], [0,3],…, [0,8]; [1,2], [1,3], etc. We chose all clusters from all intervals that have a mean fit > 0.8 inside the cluster according to the OVL kernel, resulting in 89 clusters across various time windows. Furthermore, some clusters represented exact subwindows of other clusters, which we pruned. This resulted in 43 final clusters, which contain 49 of the 78 differential genes (see S1 Table for names, descriptions and accession numbers of these 49 genes). Hence, 29 genes were not clustered and remain singletons, representing a 37% outlier ratio. The 43 clusters are described on a general level in Fig 3. There are 31 clusters of 2 genes, 11 clusters of 3 genes and 1 cluster of 4 genes. Fig 3A displays the visualization of the cluster cascade throughout the time course. Clusters have durations of 2 days to 11 days. About half of the clusters have durations of 4 days or less (10 clusters of 2 days, 8 of 3 days and 4 of 4 days) while the others display durations of more than 4 days (4 clusters of 5 days, 3 of 6 days, 5 of 7 days, 4 of 8 days, 2 of 9 days, 2 of 10 days and 1 of 11 days). The 43 clusters can also be directly visualized in Fig 4. The clusters represent expression profiles of varying time window lengths, and take into account both the profile's expected dynamics as well as its variance, or uncertainty, from the learning of the GPs from noisy and sparse data. It is evident that the 78 expression profiles fall into numerous clusters, which explains the small cluster sizes.

### Transcription factor motif analysis

In the cellular response to radiation, several sensors detect the induced molecular damage, especially DNA damage, and trigger signal transduction pathways resulting in altered expression of many target genes [49, 50]. The promoters or enhancers of these genes may contain binding sites for one or more critical TFs such as NF*k*B and AP-1 [51], and a specific TF can promote the transcription of multiple genes [49, 52]. In our model, a conventional RT dose fraction (2 Gy) induced a temporary differential gene expression response in primary normal endothelial cells, as shown in our previous work [7].

**Fig 5:**
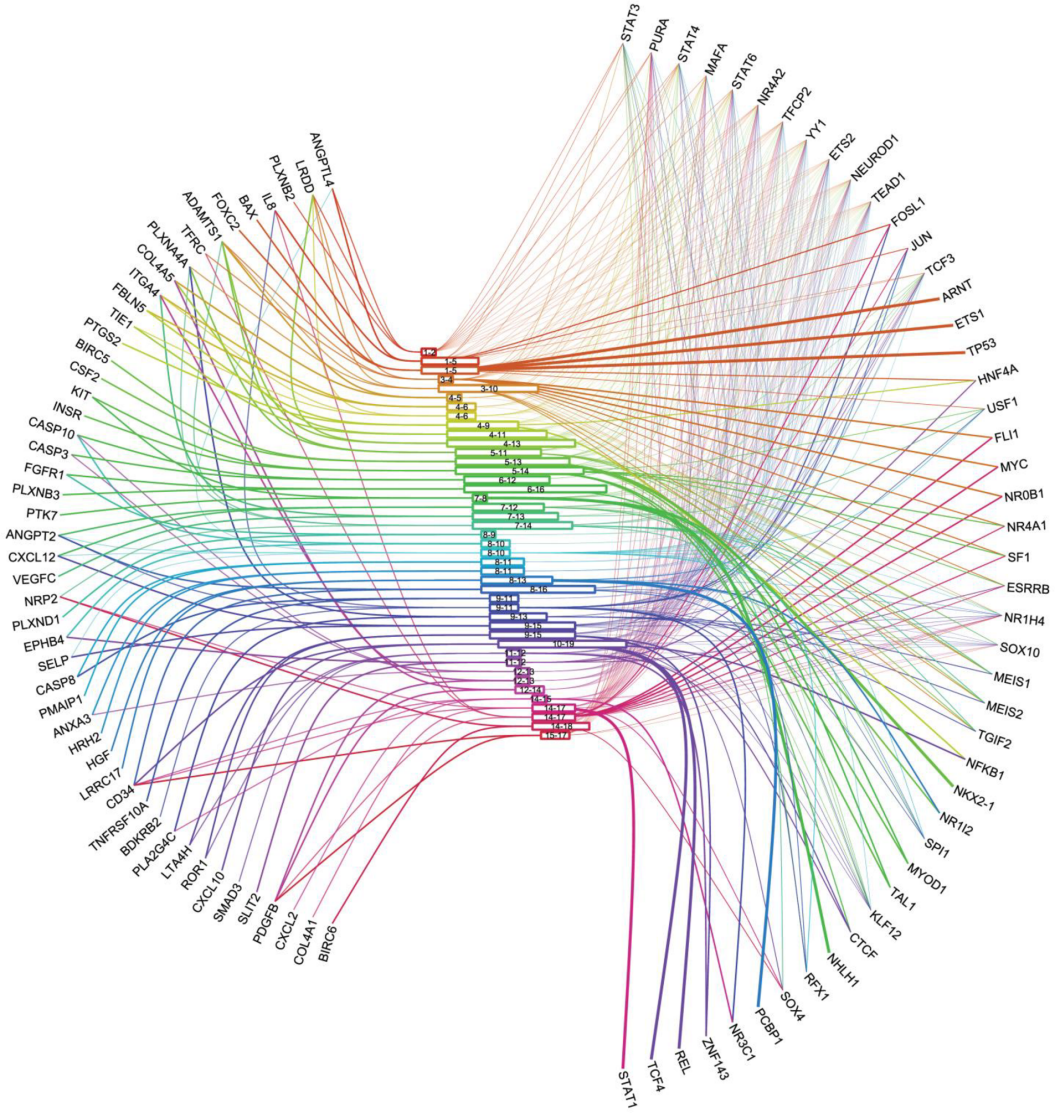
Visualization of connections between differential genes, time windows (i.e. clusters) and putative associated transcription factors. The model connects the 49 differential genes (at the left side) to the 47 TFs (at the right side) through the 43 time windows (at the center).

To gain insights into the transcriptional response of endothelial cells following irradiation, we did transcription motif analysis on our genes and tried to take advantage of our clustering method to analyze this information. We extracted all putative transcription motifs related to the original 301 genes from the MotifMap system (motifmap.ics.uci.edu) [39, 40], with a BBLS score of at least 1 and an FDR level of 0.1. These motifs are given in S2 Table. We furthermore filtered from these motifs only those that applied to the 83 differential genes. These are displayed in S3 Table. These motifs finally corresponded to 52 TFs (S3 Table) that were searched with the software Pathway Studio Web Mammal version 11.2 by querying the Mammal (ChemEffect; DiseaseFx; CellEffect) version 11.2.5.6 (Updated May 28, 2016) database from Elsevier (www.elsevier.com/pathway-studio) [46]. In the end, this resulted in 47 distinct associated TFs retrieved from this database (see S4 Table for names, descriptions and accession numbers). Table 1 summarizes the characteristics of the 43 clusters and predicted TFs found with the MotifMap system (see also S5 Table for a complete list of the clusters, motifs and TF names from MotifMap and Pathway Studio). We additionally plotted an eye diagram [41] where we matched 1) the 49 clustered genes to 2) the 43 time window clusters to 3) the 47 associated TFs found through MotifMap (Fig 5). Here, for each cluster, we connected to it all TFs that had binding sites on at least one of the genes of the clusters. The eye diagram allows the visualization of the data in one diagram.

**Table 1:** Final list of clusters, genes, and associated transcription factors.

**Clusters**	**Time window (days)**	**Duration (days)**	**Genes**	**Predicted transcription factors (names from Pathway Studio)**
**1**	"1–2"	2	ANGPTL4, PIDD1, PLXNB2	ETS12, MAFA, NEUROD1, NR4A2, PURA, STAT3, STAT4, STAT6, TEAD1, TFCP2, YY1
**2**	"1–5"	5	ANGPTL4, CXCL8	FOSL1, JUN, NEUROD1, NR4A2, PURA, STAT3, TCF3, TEAD1, TFCP2, YY1
**3**	"1–5"	5	BAX, FOXC2	ARNT, ETS1, ETS11, ETS12, HNF4A, NEUROD1, NR4A2, STAT4, TCF3, TEAD1, TFCP2, TP53, USF1, USF11
**4**	"3–4"	2	ADAMTS1, PIDD1, TFRC	ESRRB, ETS12, FLI1, MYC, NEUROD1, NR0B1, NR1H4, NR4A1, NR4A2, SF1, SOX10, STAT4, STAT6, TEAD1, TFCP2, YY1
**5**	"3–10"	8	ADAMTS1, PIDD1, PLXNA4	ETS12, MEIS1, MEIS2, NEUROD1, NR4A2, SOX10, STAT3, STAT4, STAT6, TEAD1, TFCP2, TGIF2, YY1
**6**	"4–5"	2	ADAMTS1, COL4A5	MAFA, NEUROD1, SOX10, STAT4, TEAD1, TFCP2, YY1
**7**	"4–6"	3	FBLN5, ITGA4	TEAD1, STAT6, SOX10, NEUROD1, MAFA
**8**	"4–6"	3	ITGA4, PIDD1, TIE1	ETS12, MAFA, NEUROD1, NR4A2, SOX10, STAT4, STAT6, TEAD1, YY1
**9**	"4–9"	6	FBLN5, PTGS2	ETS12, HNF4A, NFKB1, PURA, SOX10, STAT6, TEAD1, YY1
**10**	"4–11"	8	COL4A5, TIE1	MAFA, NEUROD1, NR4A2, SOX10, TFCP2, YY1
**11**	"4–13"	10	ADAMTS1, PIDD1	ETS12, NEUROD1, NR4A2, SOX10, STAT4, STAT6, TEAD1, TFCP2, YY1
**12**	"5–11"	7	ADAMTS1, PLXNA4	MEIS1, MEIS2, NEUROD1, NR4A2, SOX10, STAT3, STAT4, TEAD1, TFCP2, TGIF2
**13**	"5–13"	9	BIRC5, CSF2	ETS12, MAFA, NEUROD1, NKX2-1, NR1I2, SOX10, SPI1, STAT3, STAT4, TEAD1, YY1
**14**	"5–14"	10	INSR, KIT	ESRRB, KLF12, MYOD1, NEUROD1, NR4A1, NR4A2, SF1, STAT3, TAL1, TCF3, TEAD1, TFCP2, USF1
**15**	"6–12"	7	CASP10, CASP3	STAT3
**16**	"6–16"	11	FGFR1, PLXNB3	CTCF, ETS12, KLF12, TCF3, TEAD1, YY1
**17**	"7–8"	2	KIT, PTK7	KLF12, MYOD1, NEUROD1, NHLH1, STAT3, TAL1, TCF3, TEAD1, TFCP2, YY1
**18**	"7–12"	6	ANGPT2, CXCL12	ETS12, KLF12, NEUROD1, NR1H4, RFX1, SPI1, STAT6, TCF3, TEAD1, TFCP2, YY1
**19**	"7–13"	7	ITGA4, VEGFC	MAFA, NEUROD1, USF1
**20**	"7–14"	8	ADAMTS1, NRP2, PLXNA4	ETS12, MEIS1, MEIS2, NEUROD1, NR4A2, SOX10, SOX4, STAT3, STAT4, TEAD1, TFCP2, TGIF2, YY1
**21**	"8–9"	2	FGFR1, PLXND1	ETS12, TCF3, TEAD1
**22**	"8–10"	3	CASP10, EPHB4	MAFA, TEAD1
**23**	"8–10"	3	ANGPT2, ANGPTL4, CXCL12, SELP	ETS12, KLF12, NEUROD1, NR1H4, PURA, RFX1, SOX10, SPI1, STAT3, STAT6, TCF3, TEAD1, TFCP2, USF1, YY1
**24**	"8–11"	4	CASP8, PMAIP1	ETS12, NEUROD1, STAT4, TFCP2
**25**	"8–11"	4	ANXA3, HRH2	MAFA, NEUROD1, TEAD1, TFCP2, YY1
**26**	"8–13"	6	HGF, LRRC17	ETS12, JUN, NR1H4, NR1I2, PCBP1, SPI1, TEAD1
**27**	"8–16"	9	ANGPT2, CD34	ETS12, NEUROD1, NR1H4, NR4A2, RFX1, SOX10, SPI1, STAT6, TEAD1, TFCP2, YY1
**28**	"9–11"	3	CASP8, TNFRSF10A	ETS12, STAT4
**29**	"9–11"	3	ANGPT2, BDKRB2, CXCL8	ETS12, FOSL1, JUN, KLF12, NEUROD1, NR3C1, NR4A2, PURA, RFX1, SOX10, SPI1, STAT4, STAT6, TCF3, TEAD1, YY1
**30**	"9–13"	5	PLA2G4C, PLXNA4	MEIS1, MEIS2, NR4A2, STAT3, TEAD1, TGIF2
**31**	"9–15"	7	CXCL12, SELP	ETS12, KLF12, NEUROD1, NR1H4, SOX10, STAT6, TCF3, TEAD1, TFCP2, USF1, YY1
**32**	"9–15"	7	LTA4H, ROR1	CTCF, ETS12, KLF12, NEUROD1, NR4A2, PURA, SOX10, STAT4, TCF3, TEAD1, ZNF143
**33**	"10–19"	10	CD34, CXCL10	ETS12, NEUROD1, NFKB1, NFKB1, NR1H4, NR4A2, REL, SOX10, SOX4, STAT6, TCF4, TEAD1, TFCP2, YY1
**34**	"11–12"	2	LTA4H, ROR1, SMAD3	CTCF, ETS12, KLF12, NEUROD1, NR4A2, PURA, SOX10, STAT4, TCF3, TEAD1, ZNF143
**35**	"11–12"	2	EPHB4, SLIT2	ESRRB, HNF4A, MAFA, NEUROD1, NR1H4, NR4A2, STAT6, TCF3, TEAD1, TFCP2, YY1
**36**	"12–13"	2	ANXA3, CASP10, CASP3	MAFA, NEUROD1, STAT3, TEAD1, TFCP2, YY1
**37**	"12–13"	2	COL4A5, PDGFB	ETS12, MAFA, NEUROD1, NR4A2, PURA, TEAD1, TFCP2, YY1
**38**	"12–14"	3	ITGA4, PDGFB	ETS12, MAFA, NEUROD1, NR4A2, PURA, TEAD1, YY1
**39**	"14–15"	2	CD34, CXCL2, PLA2G4C	ETS12, NEUROD1, NR1H4, NR3C1, NR4A2, SOX10, STAT6, TEAD1, TFCP2, YY1
**40**	"14–17"	4	CD34, COL4A1, NRP2	ETS12, NEUROD1, NR1H4, NR4A2, PURA, SOX10, SOX4, STAT1, STAT6, TEAD1, TFCP2, YY1
**41**	"14–17"	4	CXCL8, PDGFB, TFRC	ESRRB, ETS12, FLI1, FOSL1, JUN, MAFA, MYC, NEUROD1, NR0B1, NR1H4, NR4A1, NR4A2, PURA, SF1, TCF3, TFCP2
**42**	"14–18"	5	BIRC6, NRP2	ETS12, NEUROD1, NR4A2, PURA, SOX4, TEAD1, TFCP2, YY1
**43**	"15–17"	3	CD34, PDGFB	ETS12, MAFA, NEUROD1, NR1H4, NR4A2, PURA, SOX10, STAT6, TEAD1, TFCP2, YY1

### Biological perspectives from gene clustering and predicted transcription factors

Using the diagram of Fig 3A, we grouped the different clusters at each day (from 1 to 21 days) or each time window (1–4 d, 4–7 d, 7–10 d, 10–14 d, 14–17 d and 17–21 d) post-irradiation. The number of clusters per day or per time window was plotted as a function of time, and compared to the number of differentially expressed genes per day (Fig 3B), or to the total number of genes within the clusters per day (Fig 3C). We also considered the number of predicted TFs (and also of genes) and plotted these numbers as a function of time at each day post-irradiation (Fig 3C).

**Fig 6:**
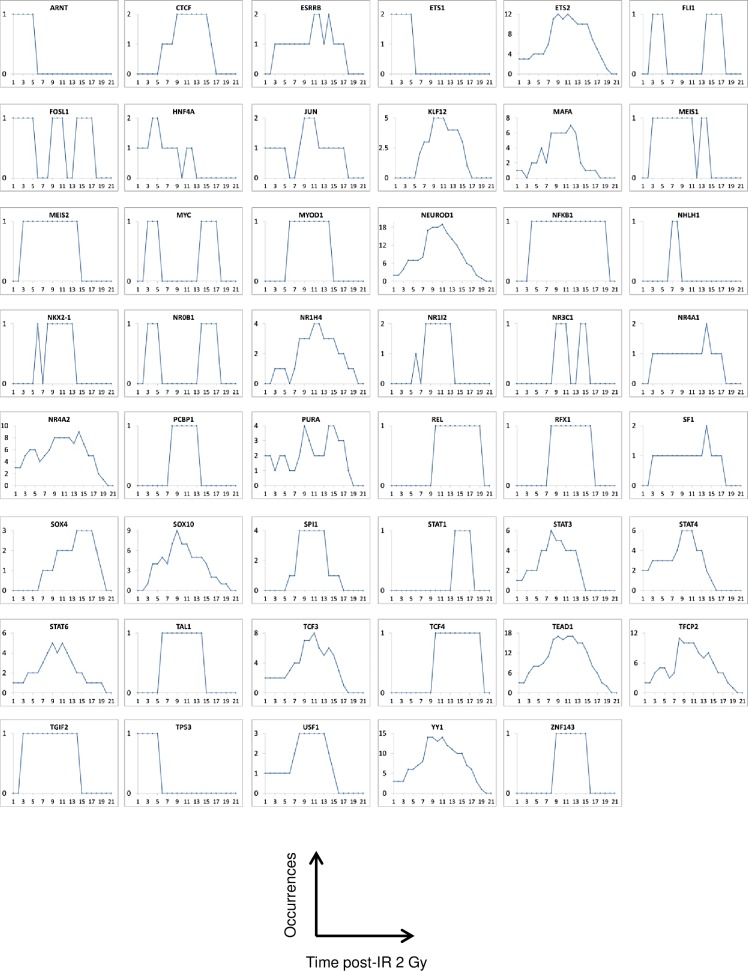
Transcription factor occurrence profiles. The number of times each TF was predicted using the MotifMap system was plotted as a function of time post-irradiation.

Considering the wealth of information given by the TFs associated with the differential genes, we assumed that the number of times TF occurred on each day or each time window post-irradiation may help to understand the response of endothelial cells to irradiation. The number of occurrences of each TF (i.e. the number of times a TF was predicted) at each day or each time window post-irradiation was determined (S6 Table) and plotted as a function of time post-irradiation (Fig 6). The absolute numbers of occurrences are quite heterogeneous from one entity to another. Several TFs have a maximum of 1 or 2 occurrences (such as ARNT, NFKB1, REL, TP53 and CTCF), while others have more than 10 occurrences (such as ETS2 and YY1) and up to a maximum of 18 occurrences for TEAD1. We considered that kinetic profiles of occurrences are likely more biologically relevant than the absolute number of occurrences since some motifs used to associate the TFs in MotifMap could be under- or over-represented. Occurrences were then normalized in the rest of the study.

**Fig 7:**
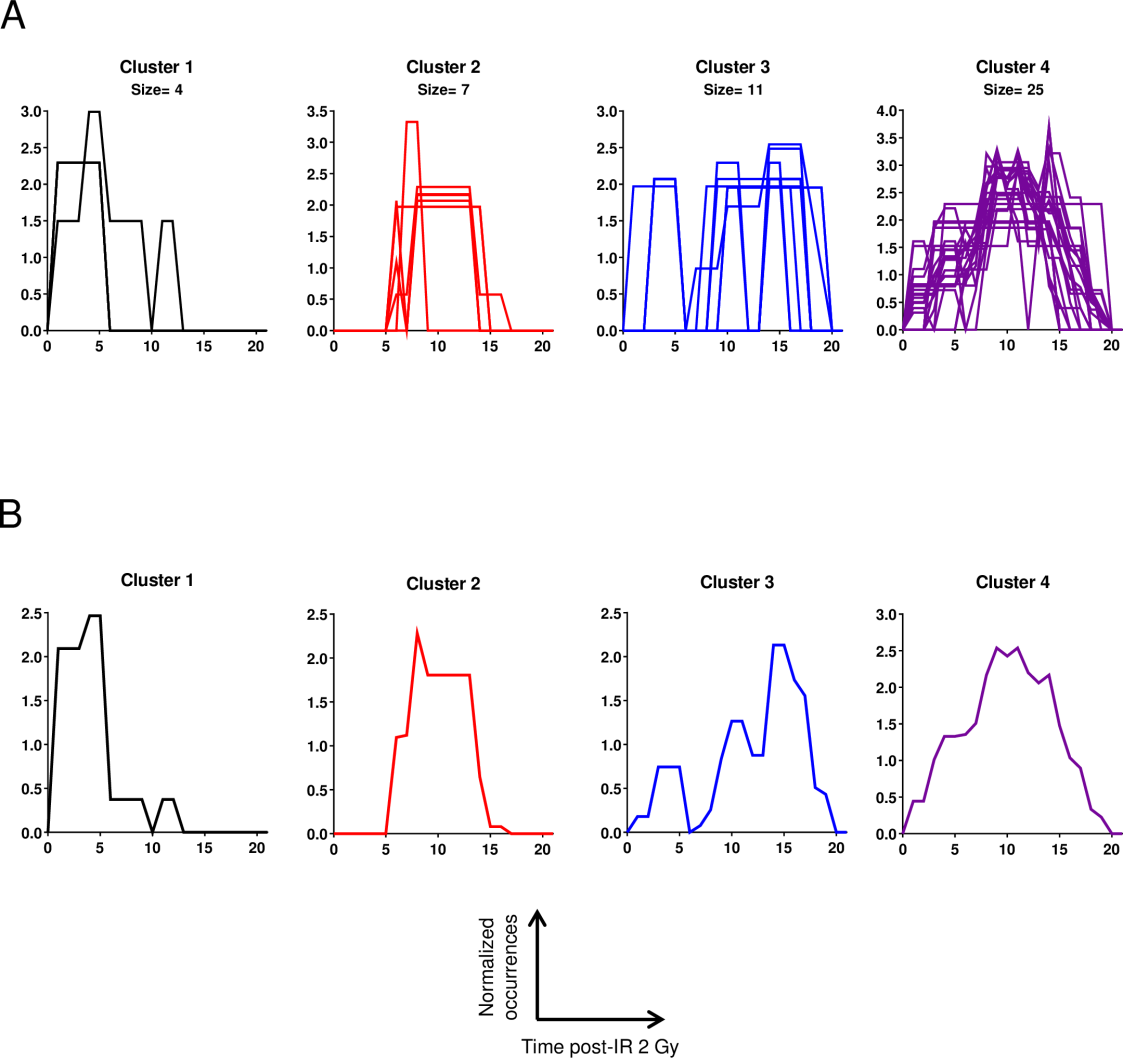
Profiles of transcription factor occurrences. (A) Occurrences of predicted associated factors were normalized, plotted as a function of time post-irradiation and clustered as described in the text, allowing the identification of four main occurrence profiles called cluster 1, cluster 2, cluster 3 and cluster 4. (B) Representative TF occurrence profiles.

To extract information from temporal profiles of occurrences, we clustered them by using functional data analysis (FDA). We obtained four main temporal profiles, i.e. i) TFs found in the early time points (early times), ii) TFs found in the middle of the time course for short periods of time (intermediate times, short periods), iii) TFs found in the late time points (late times), and iv) TFs found during long periods of time (intermediate times, long periods) (Fig 7).

A classification of the TFs according to their occurrence profiles is given in Table 2. These differences in temporal profiles are likely related to the observed differences of the temporal gene expression profiles and could provide essential biological information as discussed below.

**Table 2:** Occurrence profiles of transcription factors.

**Cluster**	**Temporal profile**	**Transcription factors**
**Cluster 1**	**Early times**	ARNT, ETS1, HNF4A, TP53
**Cluster 2**	**Intermediate times (short periods)**	MYOD1, NHLH1, NKX2-1, NR1I2, PCBP1, SPI1, TAL1
**Cluster 3**	**Late times**	FLI1, FOSL1, MYC, NR0B1, NR3C1, REL, RFX1, SOX4, STAT1, TCF4, ZNF143
**Cluster 4**	**Intermediate times (long period)**	CTCF, ESRRB, ETS2, JUN, KLF12, MAFA, MEIS1, MEIS2, NEUROD1, NFKB1, NR1H4, NR4A1, NR4A2, PURA, SF1, SOX10, STAT3, STAT4, STAT6, TCF3, TEAD1, TFCP2, TGIF2, USF1, YY1

Almost all genes were differential in the intermediate time points, as shown by the plot of the number of differentially expressed genes, which displays a maximum between 7 and 14 days post-IR (see Fig 3B). Interestingly, there are more TF occurrences in the early and late times than expected when considering the number of differentially expressed genes or the number of clusters (compare the green curve to the blue and red curves in Fig 3C). This suggests that endothelial cells may quickly activate the transcriptional machinery by modulating a few genes, which are potentially controlled by many TFs.

**Fig 8:**
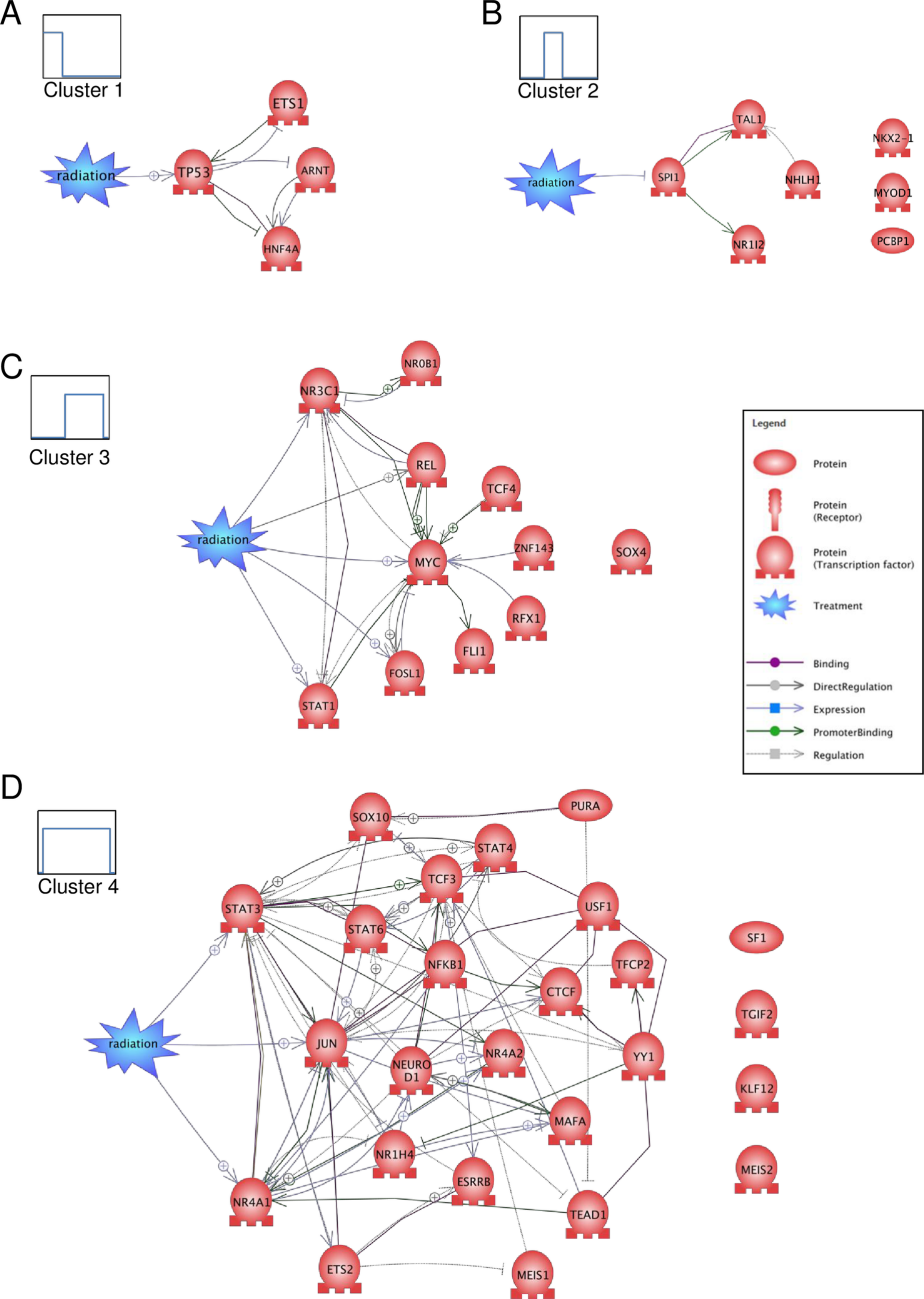
Analysis of TF and “Radiation” interaction networks. Protein networks of predicted associated TFs and the term “Radiation” as treatment were obtained for each representative profile of TFs by using the Pathway Studio software: (A), cluster 1, (B) cluster 2, (C) cluster 3 and (D) cluster 4.

Considering the TFs found in early times (ARNT, ETS1, HNF4A and TP53) (cluster 1), we established that they were interconnected and related to radiation through TP53 using the text mining algorithm of the software Pathway Studio (PS) by querying direct interactions (Fig 8A). PS identified 201 references that link radiation to TP53, 13 references that link TP53 to ETS1, 6 references that link TP53 to ARNT and 13 references that link TP53 to HNF4A. The involvement of TP53 in the response to DNA damage induced by ionizing radiation has been extensively documented [53]. In our previous investigation of the same dataset [7], we showed by bioinformatics tools (PANTHER pathway classification) that the TP53 pathway was likely activated in the early time points post-irradiation. We were able to confirm this by measuring the abundances of both TP53 and phosphorylated TP53 serine 15 proteins in HUVEC protein extracts. Here, we show that TP53 was found exclusively in the early time points by searching for associated TFs with MotifMap from the clustered genes. This result is consistent with knowledge on TP53 and helps to validate our clustering method.

Rather few TFs were found for intermediate times of short periods (cluster 2) (Fig 8B). Among these 7 TFs, only 1 TF (SPI1) was linked to radiation according to PS by only 2 references. This TF could be linked using PS to 2 other TFs. This result is not very informative of the response of endothelial cells to an irradiation dose of 2 Gy.

Concerning the late times (cluster 3), we found 11 TFs that could be involved in the response of endothelial cells to radiation (Fig 8C). Five of them were found to be linked to radiation using PS. Among them, MYC, linked to radiation by 21 references, constitutes a node of a network of almost all the other TFs. By its transcription activity at late times post-irradiation, MYC could then participate in the fate of endothelial cells, for example by participating in the resumption of cell cycle progression or cell transformation.

Finally, as regards to the 25 TFs found for a long period (cluster 4), PS shows that JUN (also known as AP-1) is strongly linked to radiation (by 31 references) and is the node of a network consisting of 14 TFs (Fig 8D). Ionizing radiation is a well-known inducer of the expression of c-jun transcription in both normal and cancer cells [50, 51], even at doses close to the RT dose fraction (1.35 Gy) [54]. Using our GP kernel gene clustering method, we therefore indirectly show here that JUN could be responsible for sustainable gene expression, in line with various publications in the field. This result also helps to validate our clustering method.

**Fig 9:**
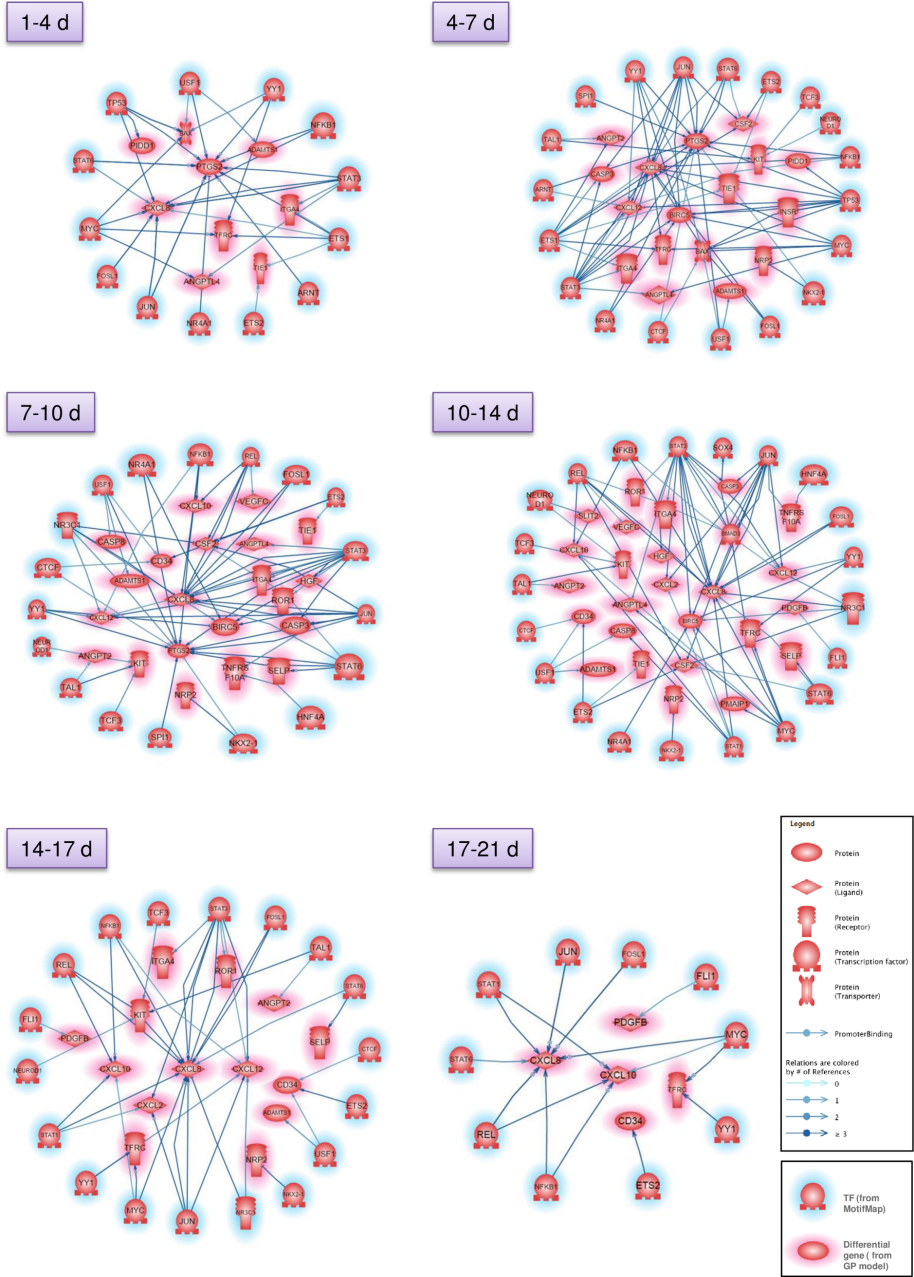
Interaction networks of differential genes and predicted transcription factors. Protein networks of differential genes and putative associated TFs were built for 6 different time windows by using the Pathway Studio software.

We then asked whether there were links between the clustered genes and the associated TFs highlighted by MotifMap. Querying promoter binding relationships with PS, we built networks between the TFs and the differential genes which were identified at the different time post-irradiation for different time windows over the 21-day post-irradiation period, i.e. 1–4, 4–7, 7–10, 10–14, 14–17 and 17–21 days (Fig 9). As shown in Fig 9, many genes and TFs were linked together, which accounted for about 40% of the input genes and 60% of the input TFs.

**Fig 10:**
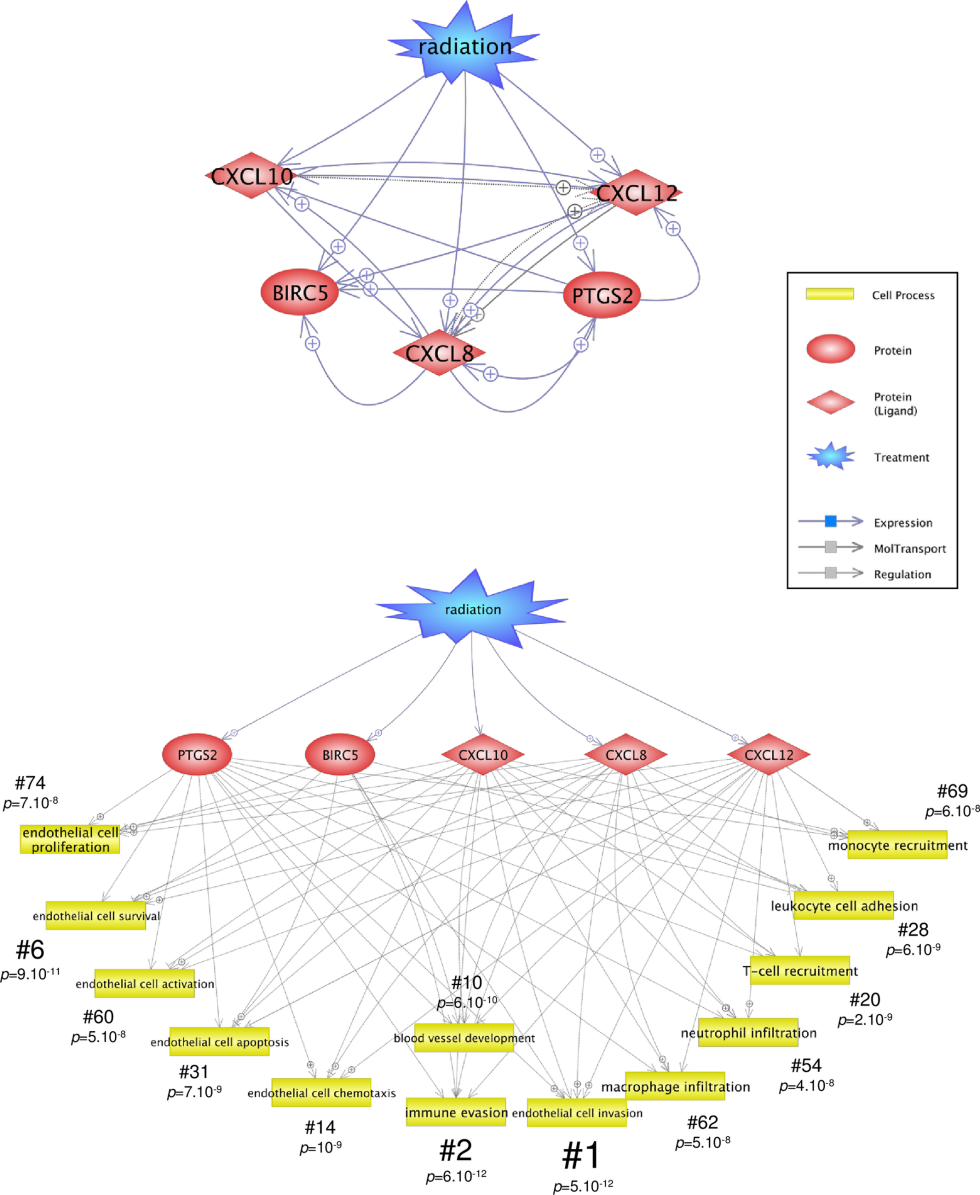
Interaction networks of the five node genes and “Radiation”. (A) Protein network of the 5 node genes BIRC5, CXCL8, CXCL10, CXCL12 and PTGS2 and the term “Radiation” as treatment was obtained using the Pathway Studio software. (B) Protein network of the 5 node genes were linked to the regulating cell processes using the subnetwork enrichment analysis module of Pathway Studio searching for proteins regulating cell processes. Fisher’s exact test *p*-values were calculated by the Pathway Studio software and are indicated for each statistically significantly identified cell process (see also S7 Table for the full list results). Ranks based on the *p*-values are indicated for each cell process (#).

Interestingly, the networks presented in Fig 9 highlight that the genes BIRC5, CXCL8, CXCL10, CXCL12 and PTGS2 are linked to several TFs, at several times post-irradiation, allowing us to consider them as molecular nodes. These genes would therefore be of interest in the understanding of the endothelial cell response to radiation. Using the subnetwork enrichment analysis module of PS searching for proteins that regulate cell processes, the pathway analysis revealed many cellular processes involved in the recruitment of immune cells and tissue infiltration, on the one hand, and processes around survival, activation, apoptosis and proliferation on the other hand (Fig 10 and S7 Table). This result is in accordance with knowledge concerning cell survival, cell proliferation and immune cell recruitment in either normal tissues or tumors [55, 56], and highlights the importance of studying these processes in response to a RT dose fraction.

### Expression of the five node genes BIRC5, CXCL8, CXCL10, CXCL12 and PTGS2 following exposure to 20 Gy

**Fig 11:**
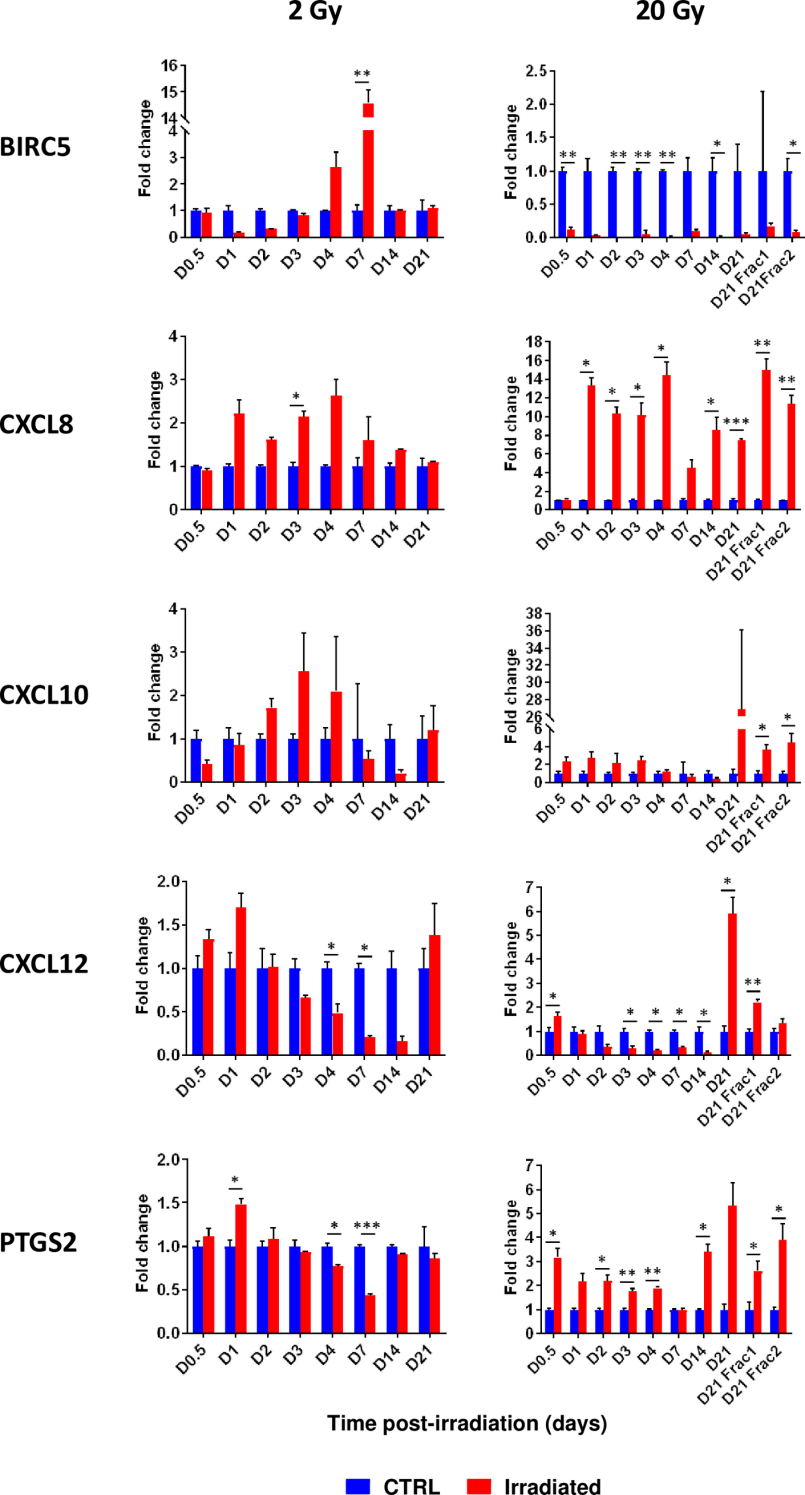
Time-course gene expression analysis of BIRC5, CXCL8, CXCL10, CXCL12 and PTGS2 following 2 and 20 Gy irradiation of HUVECs. Control and irradiated HUVEC mRNA levels of the 5 genes were measured by real-time quantitative PCR at 0.5, 1, 2, 3, 4, 14 and 21 days post-exposure at a single dose of 2 or 20 Gy, and at day 21 after the first fraction of 2 Gy (D21 Frac1) and day 21 after the last fraction of 2 Gy (D21 Frac2) for dose-fractionation experiments (mean +/- SD). Data analyzed by the two-tailed t-test and adjusted *p*-values (Benjamini-Hochberg procedure) (non-irradiated vs irradiated): *, p<0.05; **p<0.01; ***, p<0.001.

As shown in Fig 11, the expression of BIRC5, CXCL8, CXCL10, CXCL12 and PTGS2 was back to the level of the control cells after 14 to 21 days, as previously shown for expression of all other genes following 2 Gy irradiation [7]. On the contrary, we observed a sustained differential expression after 20 Gy administered either in a single dose or in 10 fractionated doses of 2 Gy (Fig 11). Interestingly, the differential temporal profiles of BIRC5 and PTGS2 were rather different between the 2 doses of radiation. BIRC5, also called survivin, is the inhibitor of apoptosis proteins [57]. The expression of BIRC5 decreased in the early times post-irradiation at 2 Gy, and then increased and finally returned to normal values, reflecting a possible wave of apoptosis or death followed by the activation of survival mechanisms. In contrast, BIRC5 was considerably down-regulated at 20 Gy, both for a single dose and for a fractionated dose, which is consistent with the low survival rate observed at these high doses [4]. Intriguingly, PTGS2 was found to be down-regulated at 2 Gy while up-regulated at 20 Gy at almost all the time points post-irradiation. PTGS2 encodes an inducible cyclooxygenase, also known as COX-2, which is the key enzyme in prostaglandin biosynthesis. Radiation generally stimulates the expression of the COX-2 protein which then mediates the production of eicosanoids such as prostaglandins and thromboxane, maintaining an inflammatory state in the tissue up to weeks after irradiation. Its action motivates the development of COX-2 inhibitors as radiation protective agents for RT [58]. At 2 Gy, down-regulation of PTGS2 may reflect a possible anti-inflammatory reaction. Although the expression of CXCL8 (i.e. IL-8), a pro-inflammatory cytokine, remained high until day 14, the decrease of PTGS2 preceded a return to normal CXCL8 expression, suggesting a causal link between these two processes in our experiments. As shown in Fig 8, there are many positive interactions between PTGS2 and CXCL8 and it has been shown several times that the inhibition of COX-2 reduces the expression of IL-8 (see for instance [59, 60]). Altogether, the results of gene expression obtained after irradiation at 2 Gy and 20 Gy call for caution when attempting to predict the effects of new radiation modalities such as stereotactic body RT (SBRT) which have made it possible to deliver one or more fractions of high-dose ionizing radiation (15–20 Gy) to tumors [61], and which is increasingly being used to treat patients [62]. Endothelial cells from both normal tissues and tumors are therefore expected to be exposed to single fractions of high radiation doses such as those used in SBRT, and *in vitro* experiments must now take into account these new practices.

## Conclusions

To gain insights into the mechanisms involved in the molecular response of endothelial cells to ionizing radiation, we applied a new GP-kernel-based clustering to gene expression time series of irradiated HUVEC cells. This method exploits the results of the previous analysis we performed by establishing a new method that combines GPs and a novel Bayesian likelihood ratio test [7]. In this previous work, we demonstrated that the method could well highlight phenomena already described in the response of cells to irradiation. Using the new approach, we go further in exploiting gene expression data. The novel proposed method introduced similarity measures for comparing GPs, allowing kernel-based supervised and unsupervised learning methods to be utilized on GPs. We additionally introduced an outlier-resistant variant of spectral clustering, which is particularly suitable for kernel-based clustering approaches. We evaluated the proposed novel kernels on a simulated clustering dataset. Using the real experimental data we generated and published earlier [7], temporal clustering over time windows, enrichment analyses and molecular pathway analysis indicate the temporal activation of biological entities and TFs by expression profile clusters.

Overall, our results highlight that a dose of 2 Gy, which corresponds to a conventional RT dose fraction, could be sufficient to activate a basic molecular program, such as cell survival, activation or cell death, and on the other hand the process of cell adhesion, which is the first step of tissue infiltration. Furthermore, based on the cluster analysis, this new method allowed us to propose putative transcription factors involved in the regulation of gene expression following radiation, and five key genes as drivers of the response to ionizing radiation in endothelial cells. The importance of these five node genes is an interesting hypothesis, whose further biological validation warrants future studies.

## Supporting information

S1 Fig The spectral k-means-clustering algorithm where the outlier-resistant k-means—clustering in the eigenspace of the graph Laplacian were used. (PPTX)

S2 Fig Comparison of the proposed kernels (Bhattacharyaa, expected likelihood, Kullback-Leibler, and overlap coefficient) within a simulated gene expression study. The OVL and BH kernels achieve a consistently high performance.
(PPTX)

S3 Fig Comparison of the proposed spectral k-means-clustering with varying outlier ratio against standard spectral k-means and spectral EM clustering algorithms within a simulated experiment. The outlier approach achieves an overall performance similar to that of standard k-means, but with higher precision and lower recall.
(PPTX)

S1 Table Complete list of the 49 differential genes found in the 43 clusters. The names, descriptions and Swiss-Prot IDs of the 49 statistically differentially expressed genes (determined by the GPR model as previously published in [7]) found in the 49 clusters of temporal expression are given.
(XLSX)

S2 Table Motifs and transcription factors associated with the 301 measured genes (MotifMap analysis). This table presents the results of the MotifMap system analysis using an FDR of 0.1. The motifs, their location with respect to the start codon and their location in the genome, as well as the predicted TFs and their Bayesian Branch Length Score (BBLS) are given for each gene (identified by their NCBI Reference Sequence) whose expression was measured in this study.
(XLSX)

S3 Table Motifs and transcription factors associated with the 78 differential genes (MotifMap analysis). This table presents the results of the MotifMap system analysis using an FDR of 0.1. The motifs, their location with respect to the start codon and their location in the genome, as well as the predicted TFs and their Bayesian Branch Length Score (BBLS) are given for each gene (identified by their NCBI Reference Sequence) whose expression was significantly statistically expressed in this study.
(XLSX)

S4 Table Complete list of the 47 transcription factors associated with the 49 differential genes. The names, descriptions and Swiss-Prot IDs of the 47 TFs predicted from the 49 differential genes using the MotiMap system are given in this table.
(XLSX)

S5 Table List of clusters, genes, transcription motifs and associated transcription factors. This table gives the names of the genes, the motifs IDs and the names (in MotifMap and their corresponding names in Pathway Studio) of the predicted TFs for each cluster of differential genes.
(XLSX)

S6 Table Occurrences of predicted transcription factors (per day and per time window). We report here the number of times each TF was respectively predicted for each day and each time window (days 1–4, 4–7, 7–10, 10–14, 14–17 and 17–21) post-irradiation. Cluster numbers are also indicated for each day and time window.
(XLSX)

S7 Table Subnetwork enrichment of BIRC5, CXCL8, CXCL10, CXCL12, PTGS2 (regulating cell processes). The table presents the result of the subnetwork enrichment of the five node genes BIRC5, CXCL8, CXCL10, CXCL12, PTGS2 searching for regulating cell processes using the Pathway Studio software. Ranks of hits are based on the *p*-values.
(XLSX)
