# Supplementary figures and images for: Temporal clustering analysis of endothelial cell gene expression following exposure to a conventional radiotherapy dose fraction using Gaussian process clustering
Source: PLoS One. 2018 Oct 3;13(10):e0204960. doi: 10.1371/journal.pone.0204960 (PMC6169916; doi:10.1371/journal.pone.0204960)

## Slide 1
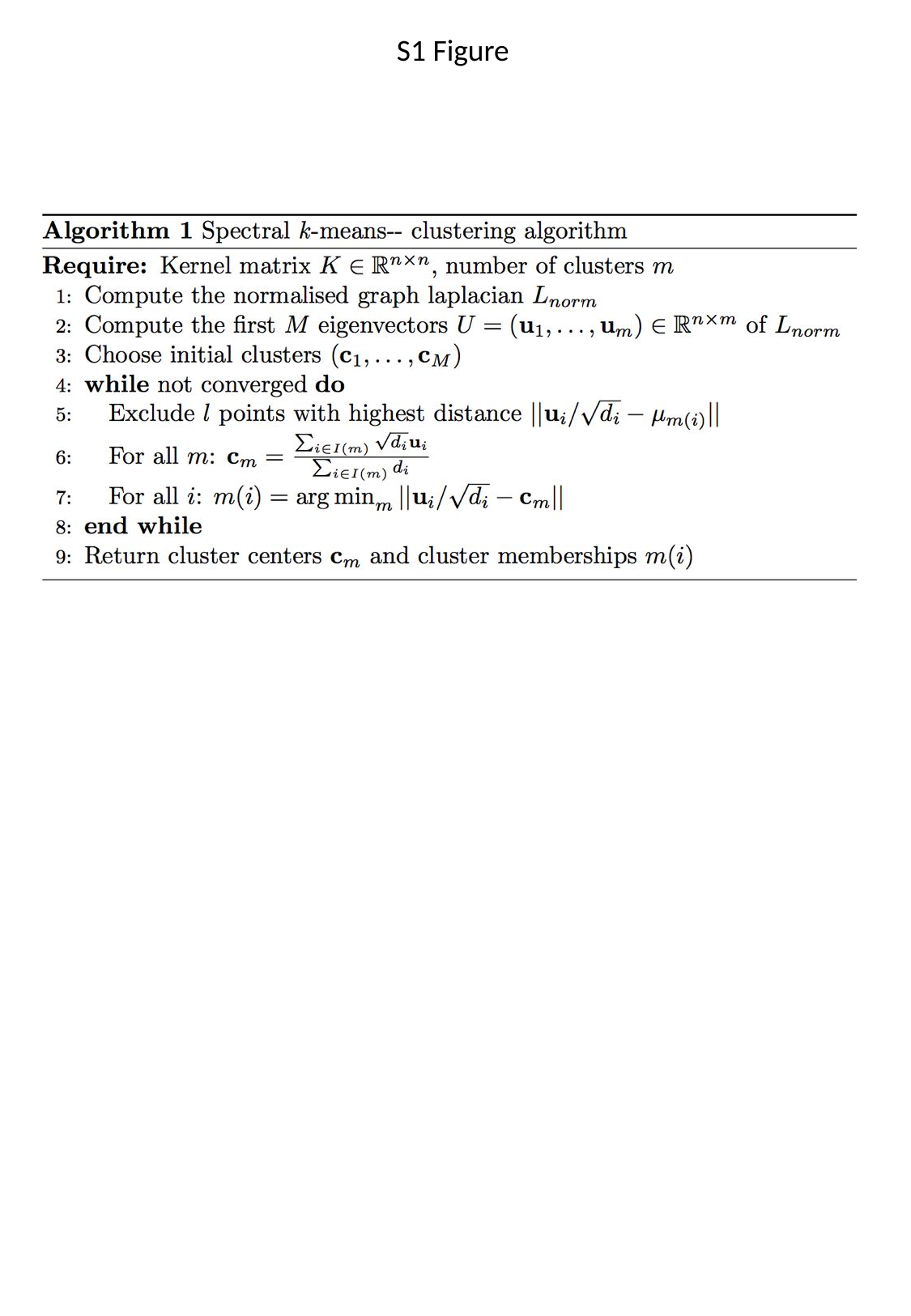

S1 Figure

Supplement: S1 Fig — (PPTX) [file pone.0204960.s001.pptx]

## Slide 1
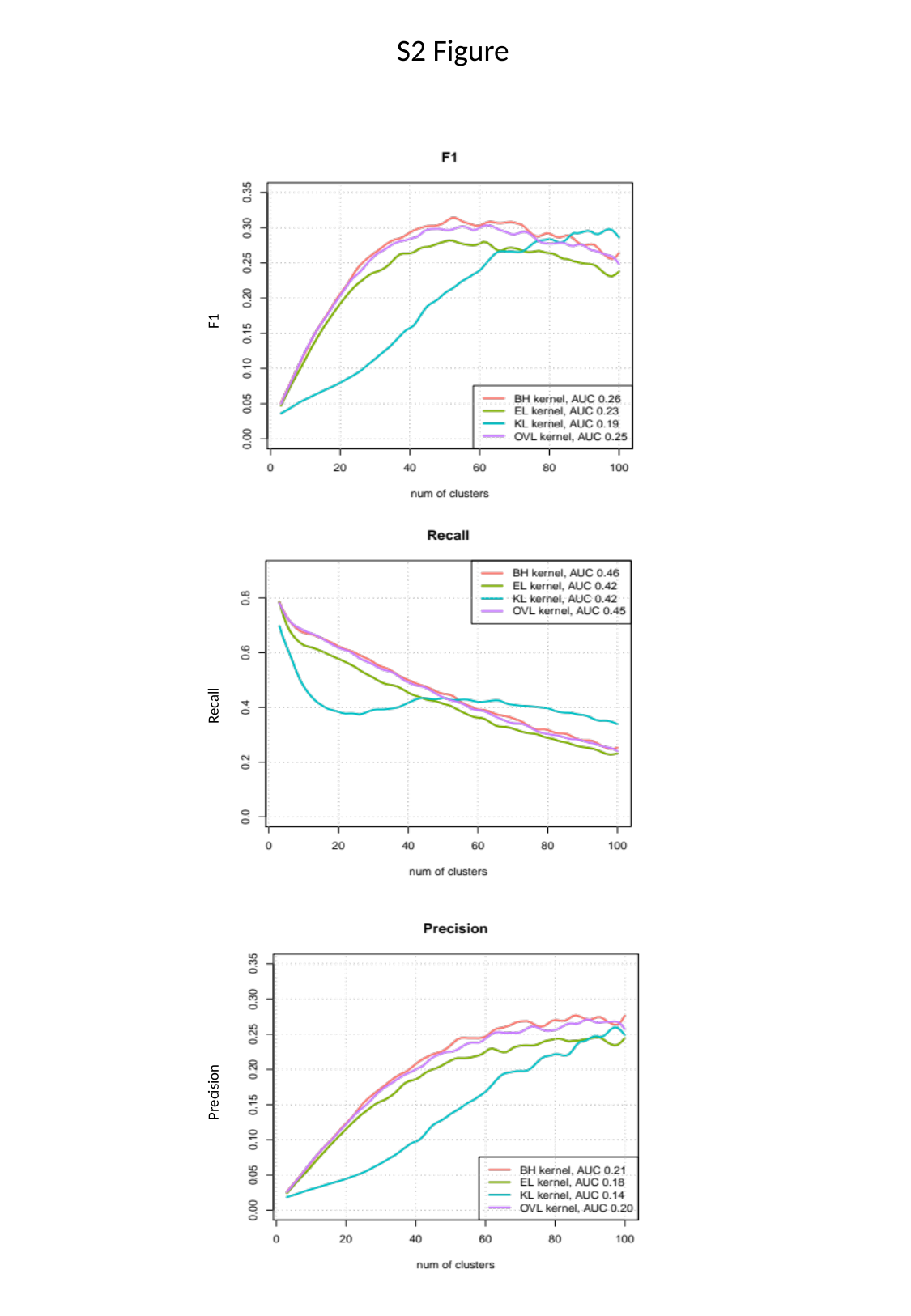

S2 Figure
F1
Recall
Precision

Supplement: S2 Fig — The OVL and BH kernels achieve a consistently high performance. (PPTX) [file pone.0204960.s002.pptx]

## Slide 1
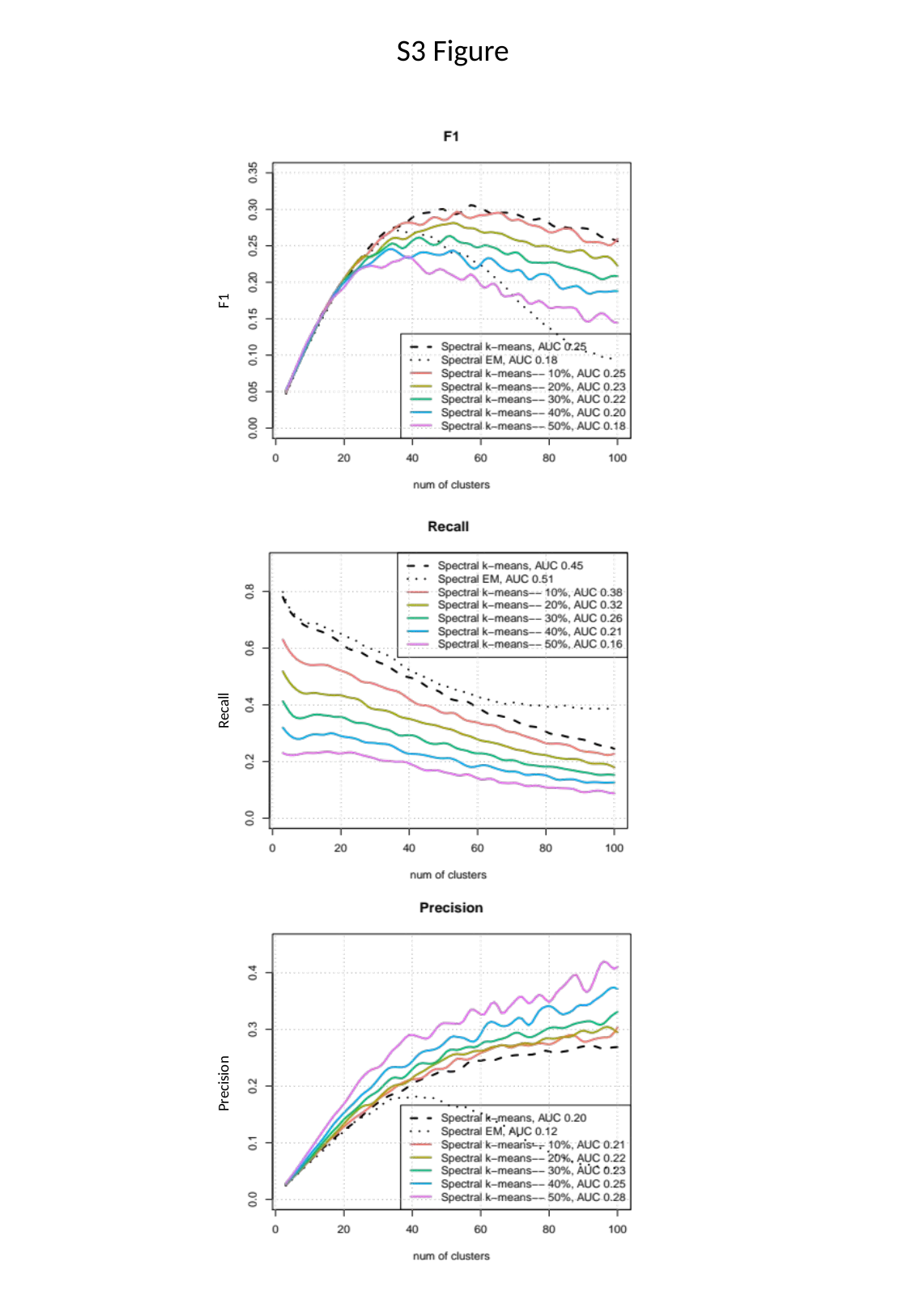

S3 Figure
F1
Recall
Precision

Supplement: S3 Fig — The outlier approach achieves an overall performance similar to that of standard k-means, but with higher precision and lower recall. (PPTX) [file pone.0204960.s003.pptx]
